# Anionically-Reinforced Nanocellulose Separator Enables Dual Suppression of Zinc Dendrites and Polyiodide Shuttle for Long-Cycle Zn-I_2_ Batteries

**DOI:** 10.1007/s40820-025-01921-y

**Published:** 2025-09-05

**Authors:** Wenhui Liu, Hong Ma, Lingli Zhao, Weiwei Qian, Bo Liu, Jizhang Chen, Yagang Yao

**Affiliations:** 1 Co-Innovation Center of Efficient Processing and Utilization of Forest Resources, College of Materials Science and Engineering, Nanjing Forestry University, Nanjing, 210037 People’s Republic of China; 2 School of Mathematics and Physics, Key Laboratory of Energy Conversion Optoelectronic Functional Materials of Jiangxi Education Institutes, Jinggangshan University, Ji’an, 343009 People’s Republic of China; 3 Shenzhen Research Institute of Nanjing University, Nanjing University, Shenzhen, 518057 People’s Republic of China; 4 National Laboratory of Solid State Microstructures, College of Engineering and Applied Sciences, Jiangsu Key Laboratory of Artificial Functional Materials, Collaborative Innovation Center of Advanced Microstructures, Nanjing University, Nanjing, 210093 People’s Republic of China

**Keywords:** Zinc-iodine batteries, Nanocellulose separators, Carboxyl functional groups, Polyiodide shuttle effect, Dendrite suppression

## Abstract

Zn-I_2_ batteries have emerged as promising next-generation energy storage systems owing to their inherent safety, environmental compatibility, rapid reaction kinetics, and small voltage hysteresis. Nevertheless, two critical challenges, *i.e.*, zinc dendrite growth and polyiodide shuttle effect, severely impede their commercial viability. To conquer these limitations, this study develops a multifunctional separator fabricated from straw-derived carboxylated nanocellulose, with its negative charge density further reinforced by anionic polyacrylamide incorporation. This modification simultaneously improves the separator’s mechanical properties, ionic conductivity, and Zn^2+^ ion transfer number. Remarkably, despite its ultrathin 20 μm profile, the engineered separator demonstrates exceptional dendrite suppression and parasitic reaction inhibition, enabling Zn//Zn symmetric cells to achieve impressive cycle life (> 1800 h at 2 mA cm^−2^/2 mAh cm^−2^) while maintaining robust performance even at ultrahigh areal capacities (25 mAh cm^−2^). Additionally, the separator’s anionic characteristic effectively blocks polyiodide migration through electrostatic repulsion, yielding Zn-I_2_ batteries with outstanding rate capability (120.7 mAh g^−1^ at 5 A g^−1^) and excellent cyclability (94.2% capacity retention after 10,000 cycles). And superior cycling stability can still be achieved under zinc-deficient condition and pouch cell configuration. This work establishes a new paradigm for designing high-performance zinc-based energy storage systems through rational separator engineering.

**Supplementary Information:**

The online version contains supplementary material available at 10.1007/s40820-025-01921-y.

## Highlights

- Straw-derived carboxylated nanocellulose separator is modified by anionic polyacrylamide to further enhance the negative charge density.
- The separator exhibits ultrathin profile and exceptional mechanical strength, as well as enabling rapid zinc ion transport.
- The separator can not only effectively inhibit zinc dendrites and parasitic reactions but also significantly suppress polyiodide shuttle via electrostatic repulsion, contributing to remarkable performance of Zn-I_2_ batteries even under high mass loadings.

**Supplementary Information:**

The online version contains supplementary material available at 10.1007/s40820-025-01921-y.

## Graphical abstract

**Figure:**
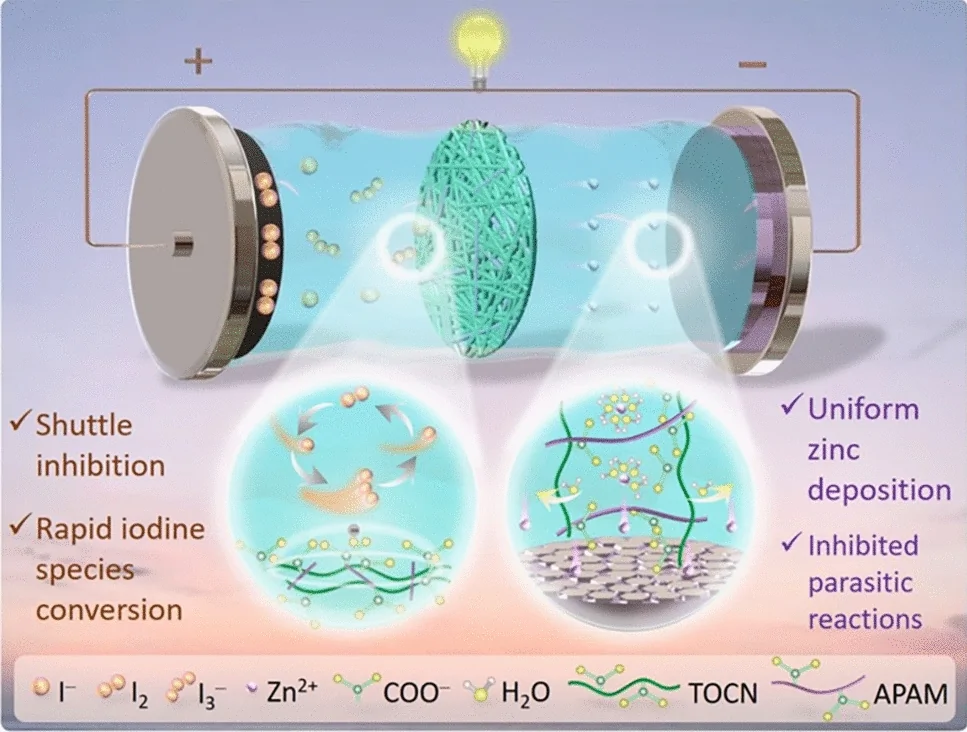

## Introduction

The increasing adoption of renewable energy has driven worldwide research efforts toward developing efficient, safe, and cost-effective energy storage systems [1, 2]. Aqueous zinc-ion batteries (AZIBs) have become a preferred system for large-scale energy storage, featuring high reliability and abundant zinc reserves [3−5]. Among them, zinc-iodine (Zn-I_2_) batteries, as an important branch of AZIBs, show virtues of low toxicity of iodine, fast redox kinetics of I^−^/I_3_^−^, and small voltage hysteresis during charge/discharge processes [6, 7]. Despite this, Zn-I_2_ batteries face the following challenges. On the one hand, the zinc anode suffers from dendritic growth induced by nonuniform electric field distribution, potentially leading to separator penetration, fast capacity decay, and compromised safety [8−10]. On the other hand, the cathode experiences polyiodide shuttle effect that triggers active material depletion, redox mediator loss, and diminished coulombic efficiency (CE) [11, 12]. The aforementioned issues have dramatically impeded the application potential of Zn-I_2_ batteries. Consequently, extensive research efforts have been devoted, including iodine host engineering [13], zinc anode protective layer design [14], electrolyte modification [15], and separator optimization [16]. In particularly, considerable research works have been conducted to develop iodine host materials, such as porous carbon [17], conductive polymers [18], metal–organic frameworks (MOF) [19], and organic frameworks [20]. While these host materials can effectively immobilize iodine and enhance electrical conductivity, their implementation presents two critical limitations: (1) their excessive usage sacrifices the overall energy density of the battery system, and (2) they fail to address the persistent challenge of zinc dendrite formation at the anode.

Separator modification represents a straightforward yet effective approach to optimize electrode interfacial environments [21, 22]. Previous studies have demonstrated that conventional glass fiber (GF) separators in AZIBs exhibit inadequate mechanical strength, nonuniform pore size distribution, and vulnerability to zinc dendrite penetration [23]. Moreover, GF separators exhibit poor ion selectivity, failing to effectively restrict the migration of polyiodide species to the anode side [24]. To address these issues, surface coating strategy has been adopted. For instance, Yang et al. modified GF separator with UiO-66-(COOH)_2_, a carboxyl-functionalized MOF, whose zinc-affinity carboxyl groups facilitate uniform zinc deposition while suppressing polyiodide shuttle, hence resulting in significantly improved electrochemical performance [25]. However, the coating strategy concurrently increases the separator thickness, ultimately compromising the battery’s energy density. Meanwhile, such strategy would exacerbate the already inflated pricing of GF separators. Consequently, designing novel separator systems that integrate cost-effectiveness, environmental sustainability, and multifunctionality has emerged as a pivotal research focus for Zn-I_2_ batteries. As the most abundant natural renewable polymer, cellulose exhibits remarkable properties, such as biodegradability, superior mechanical strength, and numerous hydroxyl groups [26]. Cellulose nanofabrication further enhances a series of properties while retaining the inherent advantages of cellulose [23]. Particularly, 2,2,6,6-tetramethylpiperidin-1-oxyl (TEMPO)-oxidized cellulose nanofiber (TOCN) owns exceptional specific surface area, outstanding mechanical strength, and abundant carboxyl groups [27].

The negative surface charges introduced by the carboxyl groups of TOCN could potentially guide uniform zinc deposition while simultaneously suppressing polyiodide migration through electrostatic repulsion. Herein, TOCN separator is constructed using straw as the raw material via a facile solution casting method, and it is further modified by anionic polyacrylamide (APAM). APAM usually serves as an effective fiber dispersant for enhancing pulp uniformity in the papermaking industry. Simultaneously, the hydrogen bonding between amide groups of APAM and hydroxyl groups of cellulose can enhance inter-fiber bonding, thereby improving paper strength [28]. Similar effect can also be achieved in the APAM-modified TOCN separator (denoted TOCN-A), contributing to a large tensile strength of 147.0 MPa. Moreover, APAM contains substantial anionic sites (primarily carboxyl groups) along its backbone, hence reinforcing the intensity of negative charges within TOCN-A separator. Experimental results show that the use of TOCN-A separator can increase ionic conductivity and Zn^2+^ ion transfer number, constrain planar Zn^2+^ ion diffusion at the Zn electrode surface, facilitate desolvation process, and reduce nucleation overpotential, thereby significantly inhibiting zinc dendrites and parasitic reactions. The Zn//Zn cell with TOCN-A separator achieves an extended cycle life of 1800 h at 2 mA cm^−2^ and 2 mAh cm^−2^, and can still offer a long lifespan under extreme conditions. And the Zn-I_2_ battery with TOCN-A separator delivers great rate capability and exceptional cycling stability.

## Experimental Section

### Preparation of SCF, TOCN, and TOCN-A Membranes

Wheat straw was initially disintegrated using a mechanical crusher, followed by ball-milling at 200 rpm for 12 h. The straw powders were treated with an aqueous solution containing 25 wt% H_2_O_2_ and 1.5 wt% NaOH under UV irradiation for 4 h, followed by washing with deionized (DI) water. After freeze-drying, straw-derived cellulose fibers (denoted SCF) can be obtained. A certain quantity of SCF was dispersed in DI water under stirring for 1 h to form a homogeneous pulp suspension, which was subsequently cast into petri dishes and then dried at 35 °C for 12 h to produce SCF membranes. 0.066 g TEMPO and 0.66 g NaBr were dissolved in 800 mL DI water under stirring while maintaining the temperature below 10 °C. Then, 4 g SCF was added to the solution and dispersed by continuous stirring. The oxidation reaction was initiated by adding 42.54 g of 10 wt% NaClO solution. The pH of the reaction mixture was maintained at approximately 10 by periodic adjustment with 0.5 M NaOH and 0.5 M HCl solutions. After 6 h, the reaction was quenched with 2 mL ethanol. The product (*i.e.*, TEMPO-oxidized cellulose fibers) was collected by centrifugation, washing with DI water, and freeze-drying. After ultrasonicating the dispersion of this product for 1.5 h, TEMPO-oxidized cellulose nanofibers (abbreviated as TOCN) can be obtained. Using TOCN as the raw material, TOCN membranes were prepared via a method analogous to that employed for preparing SCF membranes. TOCN-A membranes were fabricated by incorporating APAM into the TOCN dispersion at a mass ratio of 1 wt% prior to the casting step, while other conditions remained identical to those used for the TOCN membranes.

### Characterizations

Scanning electron microscopy (SEM) images and elemental mapping results were collected on a JEOL JSM-7600F microscope. X-ray diffraction (XRD) measurements were performed on Rigaku Ultima IV using Cu Kα radiation (*λ* = 0.1540598 nm) with a scan rate of 5° min^−1^. Fourier-transform infrared (FTIR) spectra were acquired from a Magna-IR 560 spectrometer with a wavenumber range of 4000−400 cm^−1^. Zeta potential measurements were conducted with BeNano 90 Zeta potential analyzer. Tensile stress–strain curves were obtained using a SANS UTM2502 universal testing machine. Atomic force microscope (AFM) images were acquired from a Bruker Dimensional Icon microscope. UV–vis spectra were obtained by SHIMADZU UV-2700 spectrometer.

### Electrochemical Measurements

Coin cells assembled in air were utilized for electrochemical testing. SCF, TOCN, or TOCN-A membrane was used as the separator. 2 M ZnSO_4_ aqueous solution was used as the electrolyte. Two zinc foils were utilized in Zn//Zn symmetric cells, while copper foil cathode and zinc foil anode were employed in Zn//Cu asymmetric cells. For the iodine cathode, 80 wt% I_2_@activated carbon (AC), 10 wt% Ketjen black, and 10 wt% carboxymethyl cellulose were dispersed in water to obtain a homogeneous black slurry, which was then coated onto a carbon felt. It was dried at 45 °C for 8 h and then cut into small disks with a diameter of 12 mm. The electrochemical performance of the Zn-I_2_ batteries was measured using the iodine cathode and zinc plate anode. Chronoamperometry (CA), cyclic voltammetry (CV), and electrochemical impedance spectroscopy (EIS) tests were conducted on a Bio-Logic VSP-300 multi-channel electrochemical workstation. The frequency range of EIS was set to 10^5^−0.01 Hz with an amplitude of 10 mV. Galvanostatic charge/discharge (GCD) tests were performed using a LAND CT3001A battery testing system. The Zn-I_2_ batteries were tested over a voltage range of 0.6 to 1.6 V.

## Results and Discussion

**Fig. 1:**
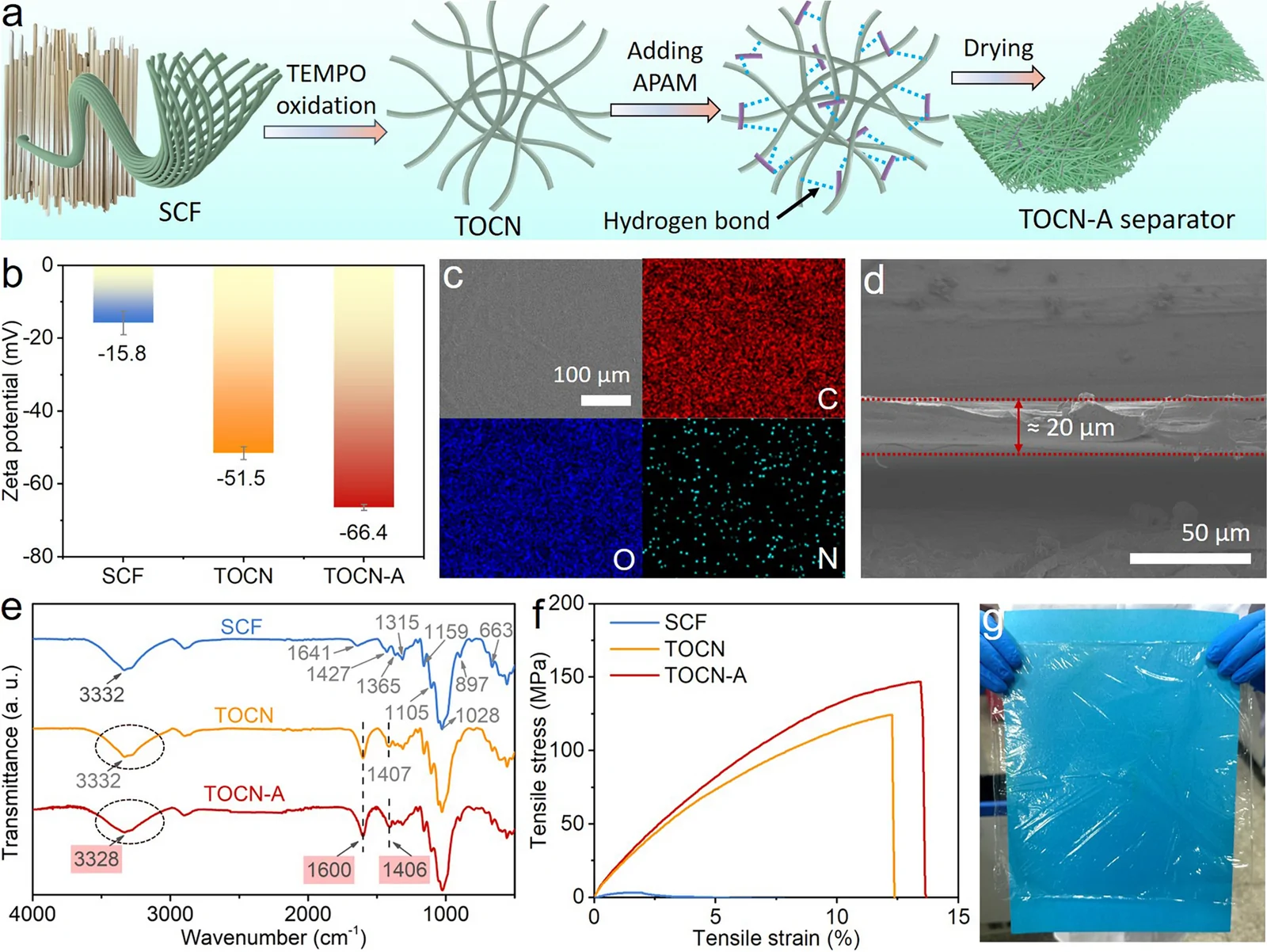
Fabrication process and characterization results. **a** Schematic illustration of the fabrication of TOCN-A separator. **b** Zeta potential values of SCF, TOCN, and TOCN-A dispersions. **c** SEM image and corresponding EDX mapping images and **d** cross-sectional SEM image of TOCN-A separator. **e** FTIR spectra and **f** stress−strain curves of SCF, TOCN, and TOCN-A separators. **g** Photograph of a large-area TOCN-A separator

Figure 1a illustrates the fabrication process of TOCN-A separator. Initially, SCF was extracted from straw. Through TEMPO-mediated oxidation, the C6 primary hydroxyl groups in cellulose chains were selectively converted to carboxyl groups [29], imparting cellulose with enhanced negative charge density. This modification facilitates subsequent depolymerization and nanosizing during ultrasonication, resulting in the formation of TOCN. Subsequently, a controlled amount of APAM was blended with TOCN, followed by solution casting and drying to fabricate the TOCN-A separator. In this configuration, TOCN and APAM can form strong hydrogen bonds, thereby enhancing mechanical properties of the complex membrane. For comparison, SCF and TOCN separators were prepared by an analogous process in the absence of APAM. The Zeta potential results in Fig. 1b show a value of − 15.8 mV for the SCF dispersion. After TEMPO oxidation, this value declines to − 51.5 mV for the TOCN dispersion, as a result of the introduction of carboxyl groups. As for the TOCN-A dispersion, the Zeta potential was further reduced to − 66.4 mV due to the addition of APAM. This trend demonstrates that TEMPO oxidation significantly increases the negative surface charge of cellulose, while the incorporation of APAM further augments the overall negative charge density. The anionic characteristic of TOCN-A cannot only enhance zincophilicity and promote the transport efficiency of Zn^2+^ cations, but also inhibit the migration of anions such as I_3_^−^ and SO_4_^2−^, hence contributing to significantly improved cycling stability of Zn-I_2_ batteries.

As illustrated in Fig. S1, the SCF separator is composed of micrometer-scale fiber bundles with a rough surface morphology, exhibiting a macroporous and structurally heterogeneous architecture. After nanosizing of cellulose, both TOCN and TOCN-A separators undergo significant changes, demonstrating dense, smooth, and transparent features with nanofibers nearly indiscernible in SEM images (Figs. S2−S4). It is also found that that the incorporation of APAM does not alter the membrane morphology. In addition, the energy-dispersive X-Ray spectroscopy (EDX) mapping images exhibit uniform distributions of C, O, and N elements throughout the TOCN-A separator (Fig. 1c), confirming homogeneous mixing of APAM with TOCN. Figure 1d reveals that the TOCN-A separator is ultrathin, being around 20 μm in thickness, which is much lower than that of conventional GF separators (200–700 μm) and various separators of zinc-based batteries in previous reports (Table S1). The use of thick separators would inevitably elevate both internal resistance and battery volume, detrimentally affecting both energy and power densities [30, 31]. Herein, the employment of TOCN-A separator with ultralow thickness can avoid such issue. The TOCN-A separator also manifests exceptional flexibility, as displayed in Fig. S5. Figure S6 compares the crystalline structures of SCF, TOCN, and TOCN-A separators. The diffraction peaks of SCF separator at 15.1°, 16.6°, 22.8°, and 34.3° correspond to the (101), (10-1), (002), and (040) planes of cellulose I, respectively [32]. As for the two other separators, they maintain almost identical diffraction peaks to SCF, confirming TEMPO oxidation and APAM addition preserve the native cellulose crystalline structure.

To analyze the influence of TEMPO oxidation and APAM incorporation on the functional groups, the separators were characterized by FTIR (Fig. 1e). The SCF separator reveals absorption bands at 3332, 1641, 1427, 1365, 1315, 1159, 1105, 1028, 897, and 663 cm^−1^, which are ascribed to − OH stretching, O − H bending, CH_2_ scissoring, C−H bending, CH_2_ rocking, C−O−C stretching, anti-symmetric in-plane stretching, C−O stretching, β−Linkage of cellulose, and OH out-of-plane bending, respectively [33]. The above bands are preserved in both TOCN and TOCN-A separators, confirming the retention of cellulose framework. Notably, new absorption bands emerge at 1600 and 1406 cm^−1^ in both TOCN and TOCN-A separators, corresponding to asymmetric and symmetric stretching vibrations of C=O of carboxyl groups, respectively [34]. This confirms successful carboxylation through TEMPO oxidation. The band corresponding to C=O symmetric stretching vibration of TOCN-A exhibits a nearly identical wavenumber to that of TOCN (1407 cm^−1^), while demonstrating a minor but discernible shift compared to APAM (1400 cm^−1^, Fig. S7). This is owing to the relatively low incorporation ratio of APAM within TOCN-A. It is also seen that the band attributed to −OH stretching experiences red shift in TOCN-A (3328 cm^−1^) compared to the other two separators (3332 cm^−1^) and APAM (3437 cm^−1^), revealing the formation of strong hydrogen bonding between TOCN and APAM [35, 36]. According to the tensile stress−strain curves in Fig. 1f, the strength of TOCN-A separator reaches 147.0 MPa, surpassing that of TOCN separator (124.4 MPa) by ~ 18% and outperforming the SCF separator (3.3 MPa) by over 44-fold, attributable to the synergistic effects of cellulose nanofibrillation and hydrogen bonding between TOCN and APAM. The tensile strength of TOCN-A separator is also superior to that of previously reported separators for zinc-based batteries (Table S1). It is worth mentioning that the solution casting method employed in this work is not restricted by high-cost equipment and complicated process, and can conveniently fabricate large-size (24 cm × 24 cm) separator (Fig. 1g). Overall, such method possesses obvious advantages over other methods such as vacuum filtration and electrostatic spinning, hence holding great application potential.

**Fig. 2:**
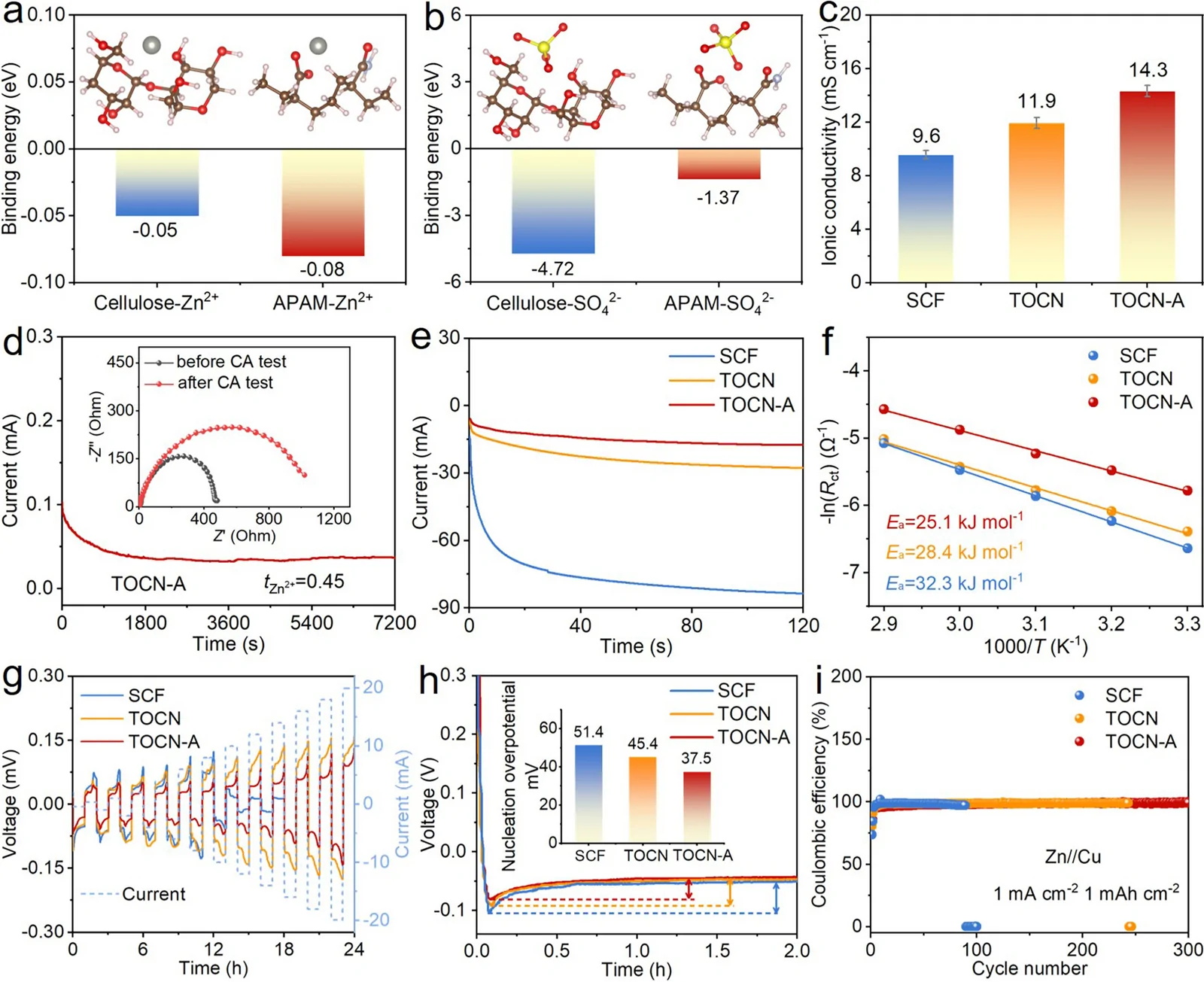
DFT calculation results and zinc stripping/plating behaviors. Binding energy values of cellulose or APAM with **a** Zn^2+^ cation and **b** SO_4_^2−^ anion. **c** Ionic conductivity values of SCF, TOCN, and TOCN-A separators. **d** CA curves and corresponding Nyquist plots, **e** CA curves at an overpotential of − 200 mV, **f** Arrhenius curves and corresponding activation energy values, and **g** CCD evaluation of Zn//Zn cells with SCF, TOCN, and TOCN-A separators. **h** Voltage–time profiles and corresponding nucleation overpotential values and **i** CE evolutions of Zn//Cu cells with SCF, TOCN, and TOCN-A separators

Density functional theory (DFT) calculations were performed to unravel the effect of APAM. As shown in Fig. 2a, the calculated results indicate that the binding energy of APAM with Zn^2+^ (− 0.08 eV) is higher than that of cellulose with Zn^2+^ (− 0.05 eV), confirming that the carboxyl groups in the APAM can enhance zincophilicity. Such strengthened coordination promotes the desolvation of [Zn(H_2_O)_6_]^2+^ complexes and enhances diffusion kinetics of Zn^2+^ ions. Besides, APAM shows a substantially weaker binding energy with SO_4_^2−^ (− 1.37 eV) compared to cellulose (− 4.72 eV) (Fig. 2b), demonstrating that employing APAM can suppress SO_4_^2−^ migration, which consequently mitigates the generation of inactive byproduct of zinc hydroxide sulfide. EIS measurements reveal superior ionic conductivity of 14.3 mS cm^−1^ for the TOCN-A separator, exceeding both TOCN separator (11.9 mS cm^−1^) and SCF separator (9.6 mS cm^−1^) (Figs. 2c and S8). According to CA and EIS test results in Figs. 2d and S9, the TOCN-A separator achieves a Zn^2+^ ion transfer number (*t*_Zn_^2+^) of 0.45, surpassing both TOCN separator (0.39) and SCF separator (0.31). This enhancement originates from the increased negative charge density in TOCN-A, which preferentially restricts SO_4_^2−^ anion mobility while simultaneously enhancing Zn^2+^ ion conduction. This can guide directional Zn^2+^ ion flux and mitigate concentration polarization of Zn^2+^ ions, thereby effectively suppressing dendritic growth and associated parasitic reactions [37, 38].

In addition, CA tests were performed at an overpotential of − 200 mV to evaluate the Zn^2+^ ion diffusion behavior (Fig. 2e). It can be seen that the polarization current with the use of SCF separator increases rapidly at the beginning and continues to rise through the whole 120 s period, indicative of sustained two-dimensional (2D) diffusion of Zn^2+^ ions that corresponds to heterogeneous zinc deposition [39]. In contrast, the polarization current increases very slowly when using TOCN and TOCN-A separators with the latter more slowly, reflecting a transition from uncontrollable 2D to stable three-dimensional (3D) diffusion of Zn^2+^ ions [40]. The 3D diffusion mode promotes the deposition of Zn^2+^ ions around their initial adsorption sites, thereby enabling smooth and uniform zinc deposition [41, 42]. EIS tests were conducted on Zn//Zn cells with three different separators across multiple temperatures to calculate desolvation activation energy (*E*_a_) (Figs. 2f and S10). The use of TOCN-A separator exhibits the smallest charge transfer impedances (*R*_ct_) across all the temperatures, and the corresponding *E*_a_ (25.1 kJ mol^−1^) is lower than that using TOCN separator (28.4 kJ mol^−1^) and SCF separator (32.3 kJ mol^−1^). These results demonstrate that the abundant negatively charged carboxyl groups in the TOCN-A separator can effectively lower the desolvation energy barrier and accelerate charge transport and zinc deposition kinetics [43, 44], correlating with the lowest onset zinc deposition potential for the use of TOCN-A separator (Fig. S11).

To assess dendrite suppression capability under high-current operation, Zn//Zn cells with SCF, TOCN, and TOCN-A separators were subjected to critical current density (CCD) tests, during which the charge/discharge time was kept at 1 h and the current was continuously increased (Fig. 2g). The results show that the cell with SCF separator presents the largest voltage hysteresis and fails at the 12th h (current density of 5 mA cm^−2^). As for the use of TOCN and TOCN-A separators, steady voltage plateaus are observed throughout all tested current densities, with the use of latter resulting in the lowest voltage hysteresis, suggesting that the TOCN-A separator is highly effective in resisting zinc dendrites and mitigating electrochemical polarization. Zn//Cu cells were also measured to evaluate the effect of separator on zinc deposition/stripping behaviors [45]. Under a current density of 1 mA cm^−2^, the nucleation overpotential value of the Zn//Cu cell with TOCN-A separator is found to be 37.5 mV, which is lower than that with TOCN separator (45.4 mV) and SCF separator (51.4 mV), as presented in Fig. 2h. This indicates that the TOCN-A separator is capable of reducing the zinc nucleation barrier, which is favorable for regulating uniform zinc deposition [46]. The long-term cyclability of Zn//Cu cells was also measured at a current density of 1 mA cm^−2^ and an areal capacity of 1 mAh cm^−2^, as shown in Fig. 2i. The Zn//Cu half cells employing SCF and TOCN separators exhibit failure behaviors after 100 and 250 cycles, respectively, indicative of unstable zinc deposition. In contrast, the Zn//Cu cell with TOCN-A separator can maintain stable CEs for over 300 cycles, along with stable voltage-capacity profiles and minimal polarization (Fig. S12), manifesting simultaneously enhanced electrochemical stability and kinetics.

**Fig. 3:**
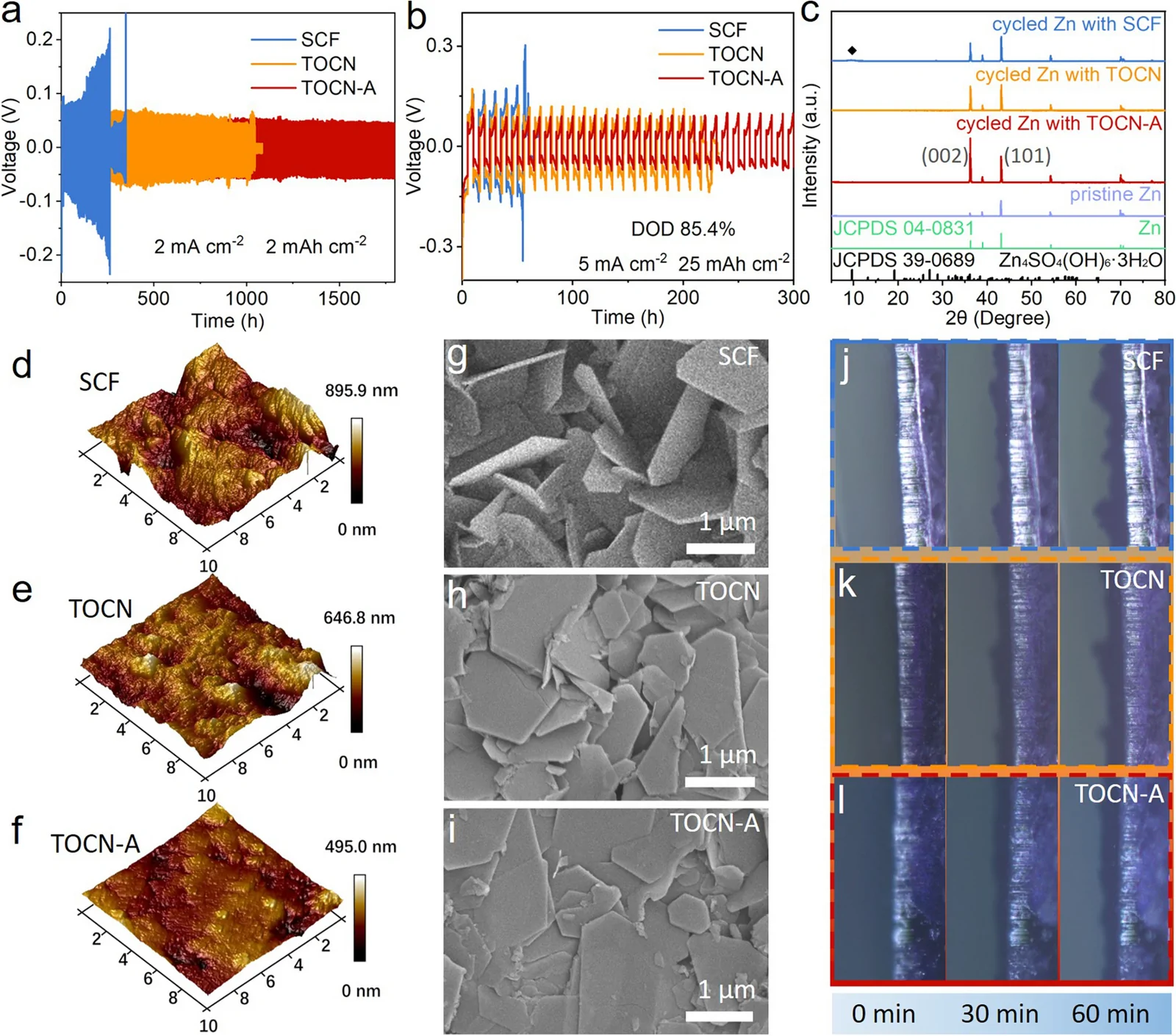
Studies on Zn//Zn cells. Cycling performance of Zn//Zn cells with SCF, TOCN, and TOCN-A separators under conditions of **a** 2 mA cm^−2^, 2 mAh cm^−2^ and **b** 5 mA cm^−2^, 25 mAh cm^−2^. **c** XRD patterns of the pristine Zn electrode and the cycled Zn electrodes using different separators. **d** − **f** AFM images and **g** − **i** SEM images of the Zn electrodes after cycling: **d**, **g** using SCF separator, **e**, **h** using TOCN separator, and **f**, **i** using TOCN-A separator. In situ optical microscopy images of the Zn electrodes during the zinc plating process at 10 mA cm^−2^: **j** using SCF separator, **k** using TOCN separator, and **l** using TOCN-A separator

Figure 3a shows cycling performance of Zn//Zn cells with different separators at 2 mA cm^−2^ and 2 mAh cm^−2^. With the use of TOCN-A separator, the Zn//Zn cell exhibits the lowest voltage hysteresis of 122 mV and achieves the longest lifespan of 1800 h. In comparison, the cells with SCF and TOCN separators exhibit larger voltage hysteresis (209 and 140 mV) and become short-circuited at 263 and 1044 h, respectively. Moreover, the Zn//Zn cell with TOCN-A separator is still able to maintain long cycling life of 1250 and 1000 h when the current density is increased to 5 and 10 mA cm^−2^, respectively, with stable voltage profiles and minimal voltage hysteresis maintained (Figs. S13 and S14). Such cyclability is significantly superior to that using SCF and TOCN separators. The cyclability of the Zn//Zn cell employing TOCN-A separator also outperforms that using various separators (including those based on nanocellulose) in previous reports, as compared in Table S1. Remarkably, even under an impressive depth of discharge (DOD) of 85.4% (5 mA cm^−2^ and 25 mAh cm^−2^), the Zn//Zn cell with TOCN-A separator still offers a lifespan a 300 h, which far exceeds that with SCF separator (56 h) and TOCN separator (223 h), as displayed in Fig. 3b. This demonstrates that the TOCN-A separator can enable exceptional reversibility in zinc deposition/stripping under extreme operational conditions [47].

After cycling tests, the Zn//Zn cells were disassembled and the cycled Zn electrodes (deposition state) were analyzed by XRD, as shown in Fig. 3c. It is found that when the SCF separator is used, in addition to the peaks of zinc metal, a peak located at 9.51° is observed, belonging to the by-product of Zn_4_SO_4_(OH)_6_·3H_2_O (JCPDS 39-0689) [48], indicative of severe side reactions at the surface of Zn electrode. Such byproduct could form a passivation layer, which would impede Zn^2+^ ion transport, increase interfacial impedance, and ultimately accelerate battery degradation [49, 50]. When using both TOCN and TOCN-A separators, only peaks belonging to the zinc metal can be detected, unveiling they are good in suppressing side reactions. Furthermore, the use of TOCN-A separator offers the highest intensity ratio of Zn(002) to Zn(101) crystalline planes (1.7), compared to that obtained with SCF separator (0.7) and TOCN separator (0.9), unambiguously demonstrating the TOCN-A separator’s capability to induce horizontally aligned zinc deposition [51, 52].

AFM and SEM characterizations were further employed to study zinc deposition behavior. As shown in Fig. 3d–f, the cycled Zn electrode with SCF separator exhibit significant unevenness with roughness of 895.9 nm, while the cycled Zn electrode with TOCN-A separator shows the flattest surface with significantly reduce roughness. According to the SEM images of Zn electrodes after cycling, almost vertically oriented flakes are observed for the use of SCF separator (Figs. 3g and S15a). Prolonged accumulation of these flakes easily leads to dendrite formation, ultimately risking separator penetration and battery short-circuit [53]. While the use of TOCN separator yields suboptimal surface topography (Figs. 3h and S15b), the overall surface flatness is much better than that using SCF separator. Satisfactorily, the introduction of TOCN-A separator endows the cycled Zn electrode with a rather smooth surface devoid of dendritic protrusions (Figs. 3i and S15c), demonstrating the effectiveness of APAM in facilitating uniform zinc deposition. In situ optical microscopy was employed to monitor zinc deposition morphology evolution during 1 h at 10 mA cm^−2^. For SCF and TOCN separators, protrusions emerge at the surface of Zn electrode within 30 min and subsequently evolve into mossy dendrites by 60 min (Fig. 3j, k). Encouragingly, the surface of Zn electrode remains flat for 60 min when using TOCN-A separator (Fig. 3l), further confirming this separator can effectively inhibit zinc dendrites.

**Fig. 4:**
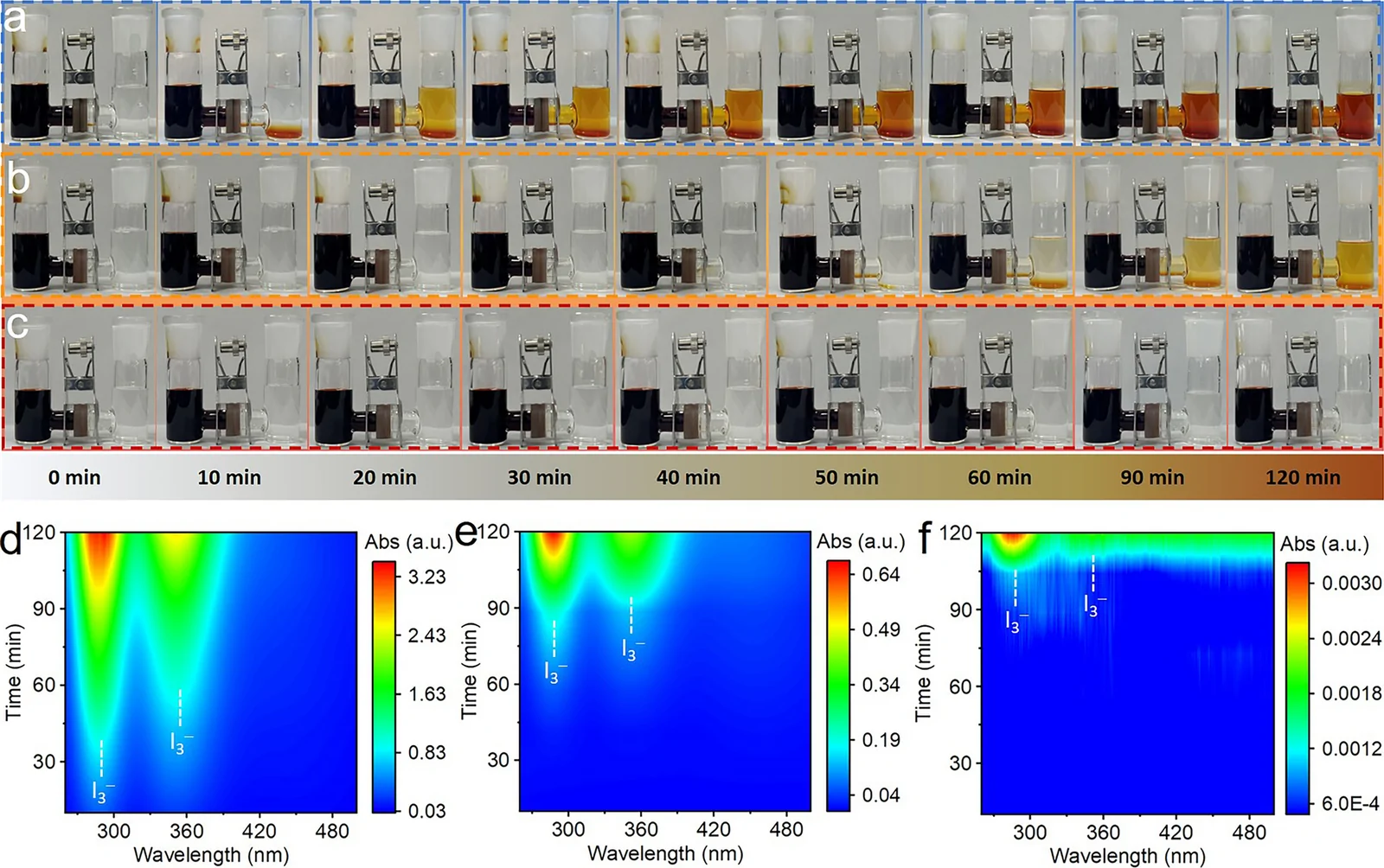
Investigations on polyiodide shuttle. Visual observations of polyiodide shuttle through **a** SCF separator, **b** TOCN separator, and **c** TOCN-A separator in H-type cell. Projections of 3D mapping images corresponding to the UV–vis spectra of I_3_^−^ concentration: **d** using SCF separator, **e** using TOCN separator, and **f** using TOCN-A separator

In order to evaluate the inhibition of polyiodide anions by different separators, visual observations of polyiodide migration were conducted using an H-shaped electrolytic cell. The left chamber of the electrolytic cell was filled with polyiodide solution, while the right chamber was filled with an equal volume of DI water, separated by SCF, TOCN or TOCN-A separator. The SCF separator permits rapid polyiodide crossover, with visible yellowish-brown coloration appearing in the right chamber within 10 min and intensifying to dark brown after 2 h (Fig. 4a). The TOCN separator delays coloration onset, exhibiting only faint yellowing after 2 h (Fig. 4b). In striking contrast, the TOCN-A separator demonstrates exceptional polyiodide confinement capability, as evidenced by the absence of visible polyiodide migration and persistent solution clarity throughout the whole period (Fig. 4c). UV–vis spectroscopy was utilized to monitor I_3_^−^ concentrations, unveiling that the implementation of SCF separator induces progressive intensification of I_3_^−^ characteristic peaks at 288 and 351 nm (Fig. 4d). After 120 min, the absorbance equates to a large concentration of 0.27 mM for I_3_^−^. The TOCN separator demonstrates moderate suppression, with delayed signal enhancement and a final I_3_^−^ concentration of 0.10 mM (Fig. 4e), representing 63% reduction compared to SCF. Most strikingly, the TOCN-A separator exhibits exceptional confinement capability. In this case, only trace I_3_^−^ signals emerge after 90 min, culminating in merely 0.05 mM concentration at 120 min (Fig. 4f), corresponding to 81% and 50% reduction versus SCF and TOCN separators, respectively. This phenomenon may be attributed to that the TOCN-A separator carries abundant carboxyl groups that make it present a more negative potential, which can greatly repel polyiodide anions.

**Fig. 5:**
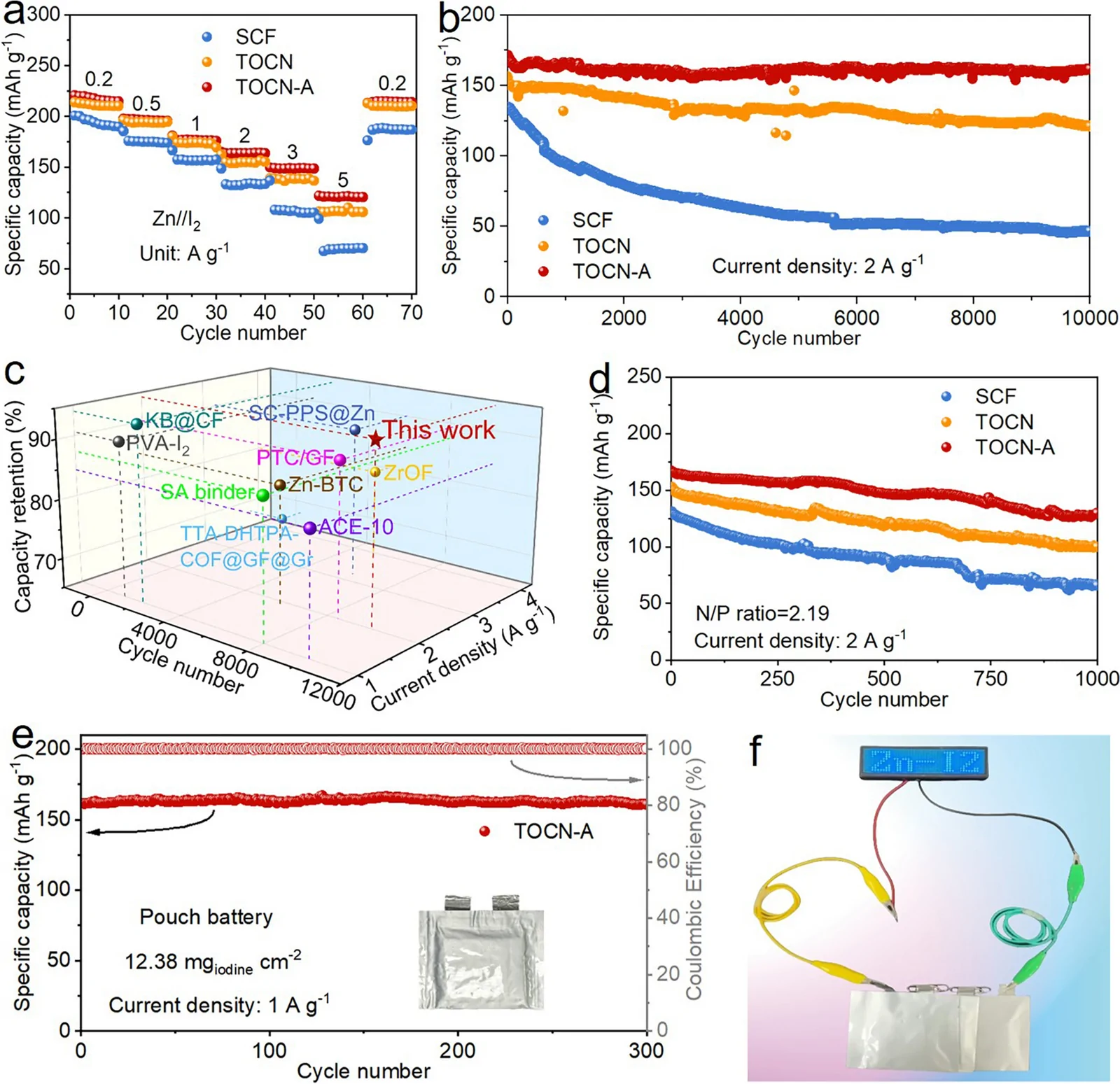
Electrochemical performance of Zn-I_2_ batteries. **a** Rate performance and **b** cycling performance at 2 A g^−1^ of Zn-I_2_ batteries with SCF, TOCN, and TOCN-A separators. **c** Comparison of the cyclability using TOCN-A separator in this work with that in previous studies. **d** Cycling performance of Zn-I_2_ batteries with SCF, TOCN, and TOCN-A separators under 2 A g^−1^ and a low N/P ratio of 2.19. **e** Cycling performance of a pouch cell with TOCN-A separator at 1 A g^−1^. **f** Photograph of utilizing pouch cells with TOCN-A separator to illuminate an LED light panel

Based on the above discussions, the TOCN-A separator demonstrates superior performance in regulating uniform zinc deposition, inhibiting side reactions, and hindering polyiodide migration. To assess its practical utility, Zn-I_2_ full batteries were assembled. After 10 cycles at 0.2 A g^−1^, the Zn-I_2_ batteries were subjected to CV tests, as shown in Fig. S16. The nearly identical redox couples in the CV curves of three batteries reveal that the separator chemistry doesn’t alter the fundamental I^−^/I_3_^−^/I_2_ redox mechanisms [6]. Additionally, the battery employing TOCN-A separator exhibits significantly higher redox peak currents compared to those with SCF and TOCN separators, manifesting superior electrochemical reactivity [17]. Notably, the TOCN-A-based battery demonstrates the lowest polarization, as evidenced by the smallest potential difference between oxidation and reduction peaks. The rate performance of these Zn-I_2_ batteries was evaluated across current densities of 0.2–5 A g^−1^ (Fig. 5a). The battery with TOCN-A separator delivers a high discharge capacity of 214.9 mAh g^−1^ at 0.2 A g^−1^. As the current density increases to 0.5, 1, 2, 3, and 5 A g^−1^, the battery maintains stable capacities of 195.5, 176.1, 163.7, 148.8, and 120.7 mAh g^−1^, respectively. Notably, when the current density is restored to 0.2 A g^−1^, the capacity recovers to 213.9 mAh g^−1^, demonstrating excellent reversibility. In comparison, the specific capacities of the batteries with SCF and TOCN separators are obviously lower. The GCD profiles of the TOCN-A battery exhibit a well-defined discharge plateau at ~ 1.25 V (Fig. S17), which is consistent with the I_2_/I^−^ redox couple [54, 55]. This stable plateau persists across all the current densities, confirming robust iodine conversion kinetics and reversibility. As for the use of TOCN separator, the battery exhibits a slight decrease in discharge plateau at 5 A g^−1^. In sharp contrast, the battery with SCF separator suffers a sharp voltage drop at 3 A g^−1^.

Figure S18 compares the long-term cycling stability of Zn-I_2_ batteries employing SCF, TOCN, and TOCN-A separators at 0.2 A g^−1^. The battery with TOCN-A separator delivers an initial discharge capacity of 219.3 mAh g^−1^, surpassing that with TOCN separator (213.2 mAh g^−1^) and SCF separator (199.6 mAh g^−1^). And the battery with TOCN-A separator can maintain 194.6 mAh g^−1^ after 1000 cycles with a high capacity retention of 88.7% and an ultralow capacity decay rate of 0.01% per cycle. Such cyclability is considerably superior to that using TOCN and SCF separators. The battery with TOCN-A separator also offers exceptional cycling stability at 2 A g^−1^ (Fig. 5b). In this respect, the battery gives a large initial capacity of 171.6 mAh g^−1^, coupled with exceptional cycling stability (94.2% capacity retention over 10,000 cycles) with an ultralow average capacity decay rate of merely 0.0058‰ per cycle. This outstanding performance starkly contrasts with the batteries with TOCN and SCF separators, which suffer from accelerated capacity fading, retaining only 77.2% and 34.4% of their initial capacities after 10,000 cycles, respectively. Such trend is also reflected by the GCD curves at varying cycle numbers (Fig. S19), with the use of TOCN-A separator resulting in the smallest voltage gaps and the most stable GCD profiles. These results clearly demonstrate that the TOCN-A separator significantly enhances iodide redox kinetics and reaction reversibility. Furthermore, comparative analysis with recent literature confirms that the TOCN-A separator achieves superior cyclability relative to recently reported Zn-I_2_ battery systems (Fig. 5c) [8, 56−63].

Notably, while employing excessively thick Zn foils in battery assembly can prevent rapid anode depletion, this approach presents significant drawbacks. The surplus zinc metal fails to contribute to the actual capacity while substantially diminishing the battery’s overall energy density [64]. Therefore, a low negative–positive electrode capacity (N/P) ratio should be used to evaluate the practical application potential [65, 66]. Herein, a thin Zn foil with 10 μm in thickness is paired with high iodine loading (16 mg cm^−2^) to realize a low N/P ratio of 2.19. As shown in Fig. 5d, the battery with TOCN-A separator enables an initial capacity of 166.9 mAh g^−1^ at 2 A g^−1^ and retains 130.5 mAh g^−1^ after 1000 cycles, significantly outperforming contrast systems over the same cycling period (TOCN: 152.3–98.4 mAh g^−1^; SCF: 130.3–65.7 mAh g^−1^). The superior performance of TOCN-A separator mainly stems from its ability in addressing the critical challenges at both zinc anode and iodine cathode. It is worth mentioning that polyiodide shuttle not only depletes cathode active material but also induces spontaneous capacity loss during battery idle periods, hence severely influencing self-discharge behavior [67]. As shown in Fig. S20, the CE of the battery with TOCN-A separator is 93.89% after 48 h of resting. This is higher than that with TOCN separator (92.74%) and SCF separator (92.14%). A pouch cell with a large iodine loading of 12.38 mg cm^−2^ was also constructed (Fig. 5e). An exceptional capacity retention of 99.4% is achieved after 300 cycles at 1 A g^−1^. Furthermore, to better demonstrate applicability, three pouch cells connected in series were used to power an LED light panel (Fig. 5f) and an electronic hygrometer (Fig. S21), providing compelling evidence for TOCN-A’s practical utility.

## Conclusions

This study develops an anionically reinforced, straw-derived carboxyl-functionalized nanocellulose separator (TOCN-A) to simultaneously address cathode and anode challenges in Zn-I_2_ batteries. The engineered separator exhibits remarkable multifunctional properties: exceptional tensile strength (147 MPa), pronounced negative surface potential (− 66.4 mV), ultrathin profile (20 μm), superior ionic conductivity (14.3 mS cm^−1^), and elevated Zn^2+^ ion transfer number (0.45). The negatively charged carboxyl groups endow the TOCN-A separator with high zinc affinity and effective SO_4_^2−^ repulsion, contributing to the regulation of Zn^2+^ ion transport, the constrained planar Zn^2+^ ion diffusion at the Zn electrode surface, facilitated desolvation process, and reduced nucleation overpotential, collectively ensuring uniform zinc deposition and significantly inhibited parasitic reactions. Experimental evaluations reveal that the TOCN-A separator considerably prolongs the cycle life of Zn//Zn cells, achieving over 1800 h at 2 mA cm^−2^ and 2 mAh cm^−2^ and 300 h under extreme conditions of 5 mA cm^−2^ and 25 mAh cm^−2^. In addition, the abundant negative charges within TOCN-A separator can effectively suppress polyiodide shuttle effect. Consequently, the Zn-I_2_ battery based on TOCN-A separator delivers a large capacity of 214.9 mAh g^−1^ at 0.2 A g^−1^, great rate capability (120.7 mAh g^−1^ at 5 A g^−1^), and excellent cycling stability (94.2% capacity retention over 10,000 cycles). And good cycling stability can still be achieved under zinc-deficient, high iodine loading, and pouch cell configurations. Overall, this study not only provides a sustainable separator material for Zn-I_2_ batteries, but also offers new insights into separator design for advanced energy storage systems.

## Supplementary Information

Below is the link to the electronic supplementary material.

Supplementary file1 (DOCX 2995 KB)
