# Supplementary material for: Anionically-Reinforced Nanocellulose Separator Enables Dual Suppression of Zinc Dendrites and Polyiodide Shuttle for Long-Cycle Zn-I2 Batteries
Source: Nanomicro Lett. 2025 Sep 5;18:59. doi: 10.1007/s40820-025-01921-y (PMC12413360; doi:10.1007/s40820-025-01921-y)
Supplement: Supplementary file 1 — Supplementary file1 (DOCX 2995 KB) [file 40820_2025_1921_MOESM1_ESM.docx]

# Supporting Information for

**Anionically-Reinforced Nanocellulose Separator Enables Dual Suppression of Zinc Dendrites and Polyiodide Shuttle for Long-Cycle Zn-I_2_ Batteries**

Wenhui Liu^1^, Hong Ma^1^, Lingli Zhao^1^, Weiwei Qian^1^, Bo Liu^2^, Jizhang Chen^1,^ * and Yagang Yao^3, 4,^ *

^1^ Co-Innovation Center of Efficient Processing and Utilization of Forest Resources, College of Materials Science and Engineering, Nanjing Forestry University, Nanjing 210037, P. R. China

^2^ School of Mathematics and Physics, Key Laboratory of Energy Conversion Optoelectronic Functional Materials of Jiangxi Education Institutes, Jinggangshan University, Ji’an 343009, P. R. China

^3^ Shenzhen Research Institute of Nanjing University, Nanjing University, Shenzhen 518057, P. R. China

^4^ National Laboratory of Solid State Microstructures, College of Engineering and Applied Sciences, Jiangsu Key Laboratory of Artificial Functional Materials, Collaborative Innovation Center of Advanced Microstructures, Nanjing University, Nanjing 210093, P. R. China

*Corresponding authors. E-mail: [chenjizhang@njfu.edu.cn](mailto:chenjizhang@njfu.edu.cn) (Jizhang Chen); [ygyao2018@nju.edu.cn](mailto:ygyao2018@nju.edu.cn) (Yagang Yao)

**S1 Experimental Section**

**S1.1 Preparation of iodine cathode material**

Iodine (I_2_) and activated carbon (AC) (1: 1 mass ratio) were thoroughly homogenized in an agate mortar, then transferred to a Teflon-lined stainless-steel autoclave for thermal treatment at 130 ℃ for 8 h. After cooling to room temperature, the autoclave was opened and reheated at 130 ℃ for 3 h to desorb excess I_2_, yielding homogeneous I_2_@AC composite.

**S1.2 Ionic conductivity measurements**

EIS tests were performed on stainless steel//stainless steel (SS//SS) symmetric cells to measure the ionic conductivity (*σ*) of SCF, TOCN, and TOCN-A separators. 2 M ZnSO_4_ aqueous solution was used as the electrolyte. The calculation formula is as follows:

$\sigma=\frac{L}{RS}$ (S1)

where *L* is the separator thickness, *S* is the contact area between the SS electrode and the separator, and *R* (Ω) is the bulk resistance obtained from the intercept of the Nyquist plots with the real axis.

**S1.3 Zn^2+^ ion transfer number measurements**

Zn//Zn symmetric cells were tested using CA and EIS tests to determine the Zn^2+^ ion transfer number (*t*_Zn_^2+^) of SCF, TOCN, and TOCN-A separators. 2 M ZnSO_4_ aqueous solution was used as the electrolyte. The calculation formula is as follows:

$t_{\mathrm{Zn}^{2+}}=\frac{I_{s}(\Delta V-I_{0}R_{0})}{I_{0}(\Delta V-{I_{s}R}_{s})}$ (S2)

where *I*_0_ and *I*_s_ represent the initial and steady-state currents, Δ*V* denotes the applied constant potential (10 mV), and *R*_0_ and *R*_s_ correspond to the initial and steady-state interfacial impedances obtained from EIS measurements, respectively.

**S1.4 *In-situ* optical microscopy observations**

*In-situ* optical microscopy images were captured by Sunny CX40M metallurgical microscope coupled with a Gaoss Union C031-5 electrochemical cell. Zn//Zn symmetric cells were employed for *in-situ* monitoring of zinc deposition process. The cell configuration consisted of two symmetric electrodes made from 200-μm-thick Zn plates (8 mm × 10 mm), separated by SCF, TOCN, or TOCN-A membrane. The cells were galvanostatically operated at 10 mA cm^−2^ for 60 min using a CT2001A battery testing system.

**S1.5 Visual observations of polyiodide migration**

Polyiodide migration was visualized using an H-type electrolytic cell. The left chamber contained 0.1 M Zn(I_3_)_2_ solution, while the right chamber contained deionized water, separated by SCF, TOCN, or TOCN-A membrane. The I_3_^−^ concentration in the right chamber was periodically monitored by UV-vis spectroscopy at specified time intervals, with concentrations quantified using a pre-calibrated standard curve.

**S2 Density functional theory (DFT) calculations**

All the calculations were performed with Vienna ab initio Simulation Package (VASP) based on the DFT [S1], within the generalized gradient approximation of the Perdew-Burke-Ernzerhof (PBE) [S2]. The interactions between ion cores and valence electrons were treated using the projector augmented wave (PAW) method [S3]. The plane-wave cutoff energy was set to be 500 eV. The cellulose and APAM surface models were simulated by a symmetric periodic slab model with consecutive slabs separated by a 15 Å vacuum layer. Throughout the simulations, van der Waals (vdW) interactions at DFT-D2 level were taken into account [S4]. The calculation formula for the interaction energy between cellulose/APAM and electrolyte ion (Zn^2+^ or SO_4_^2^^−^ ion) is as follows:

$E=E_{\mathrm{ab}}-E_{a}-E_{b}$ (S3)

where *E*_ab_ is the energy of the optimized system, *E*_a_ is the energy of isolated Zn^2+^ or SO_4_^2−^ ion, and *E*_b_ is the energy of the optimized structure of cellulose or APAM.

**S3 Supplementary Figures**


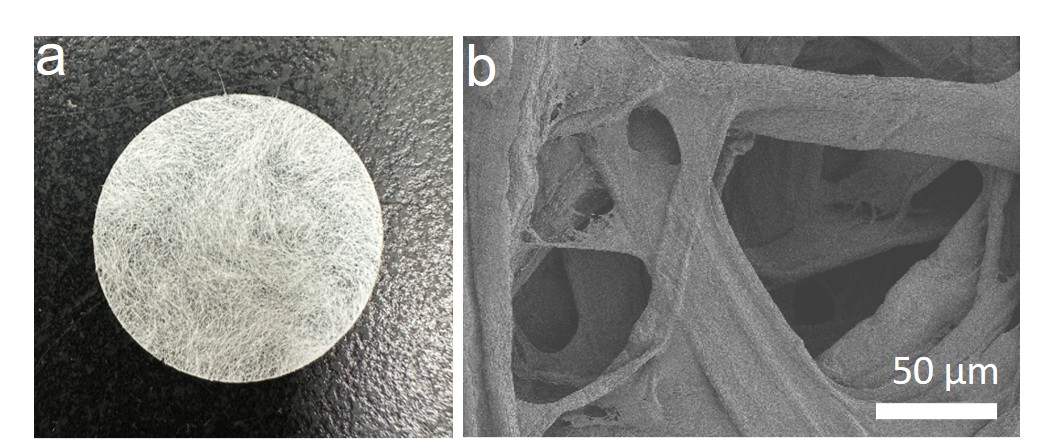


**Fig. S1** **a** Photograph and **b** SEM image of SCF separator


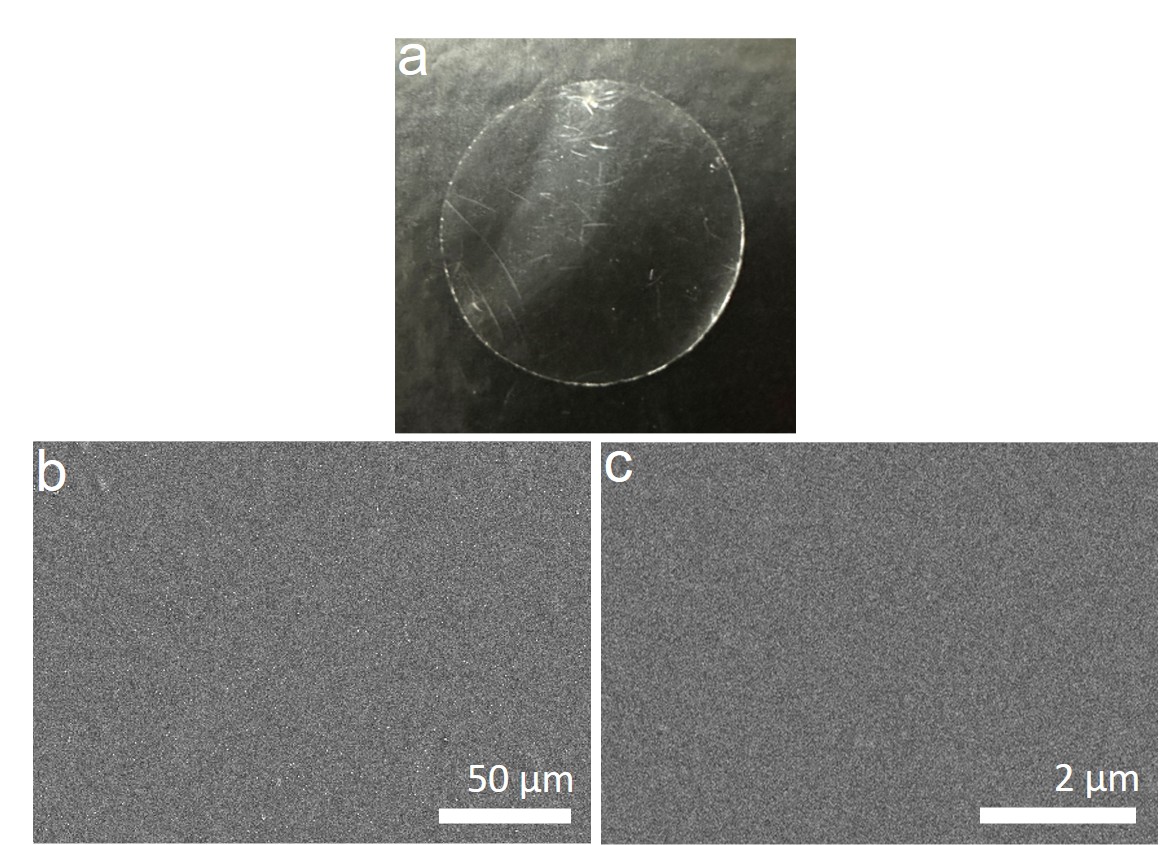


**Fig. S2** **a** Photograph and **b**, **c** SEM images of TOCN separator


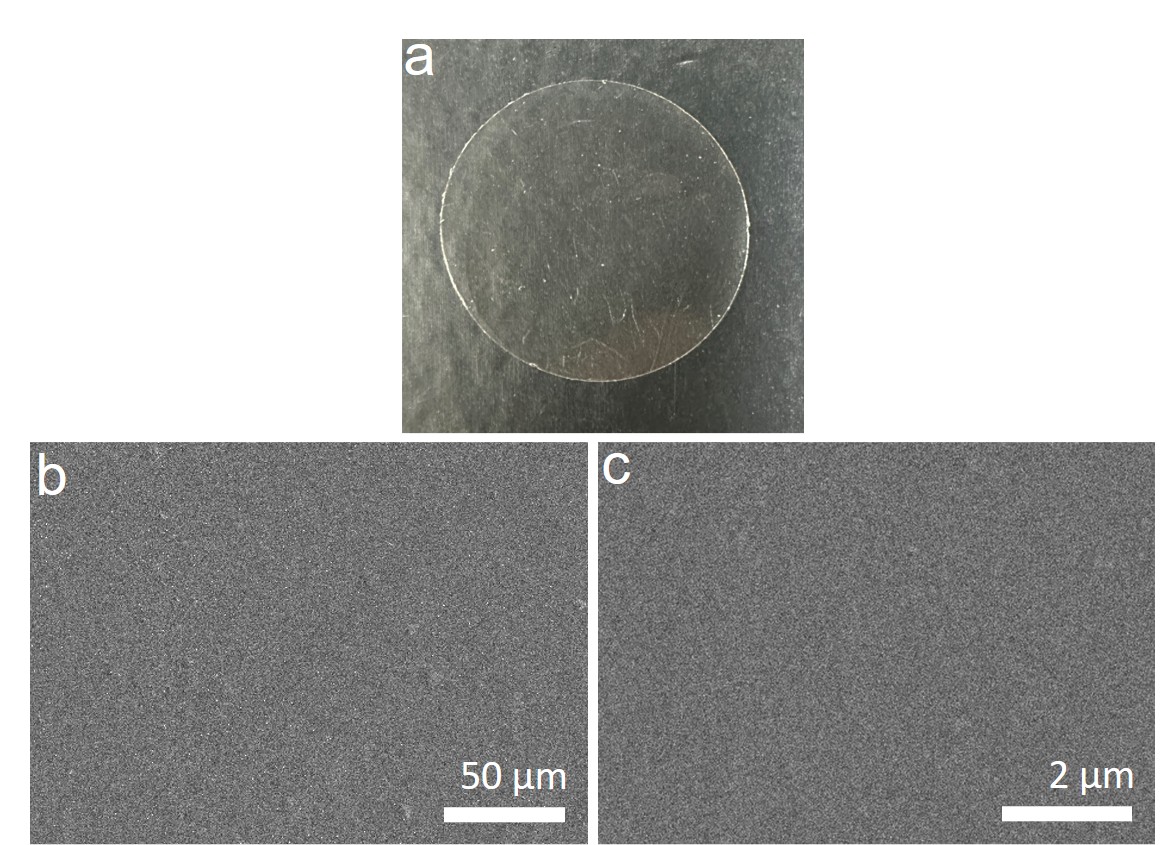


**Fig. S3** **a** Photograph and **b**, **c** SEM images of TOCN-A separator

**
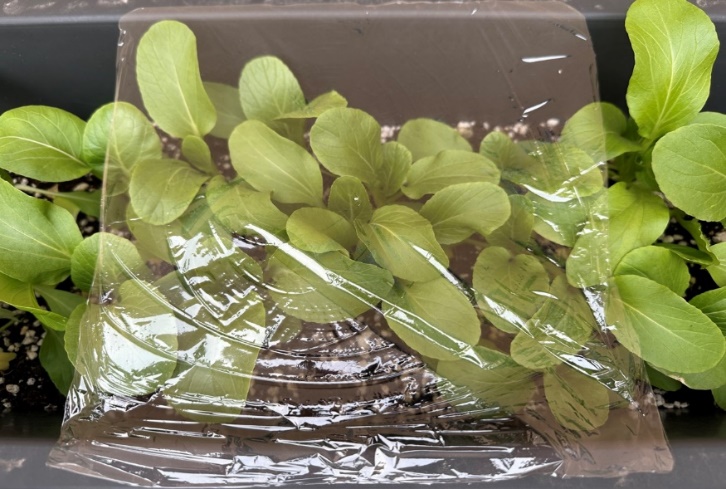
**

**Fig. S4** Photograph of TOCN-A separator, demonstrating its high transparency


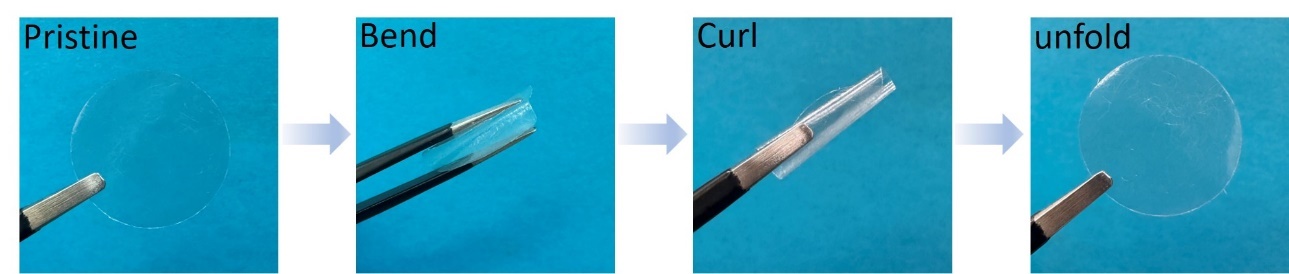


**Fig. S5** Photographs of TOCN-A separator in its pristine, bending, curling, and unfolding states

**
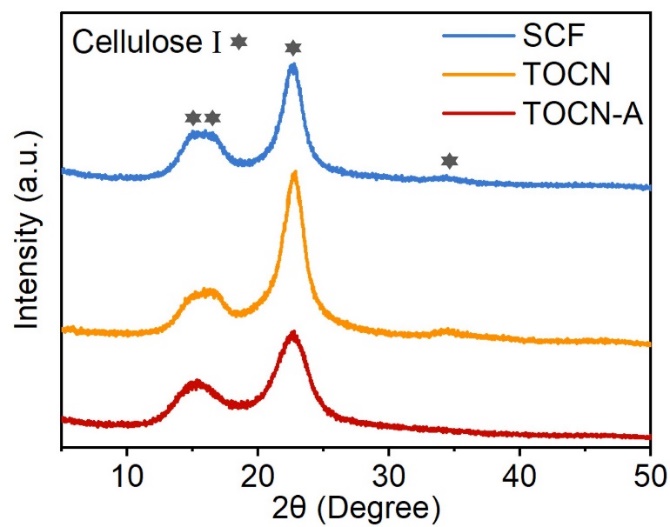
**

**Fig. S6** XRD patterns of SCF, TOCN, and TOCN-A separators

**
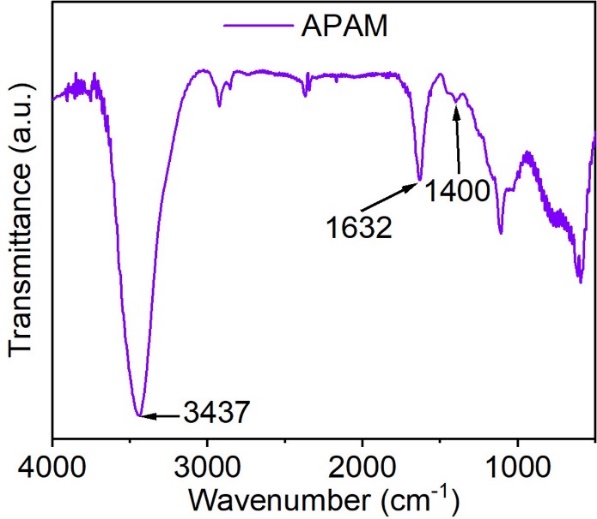
**

**Fig. S7** FTIR spectrum of APAM

The absorption bands at 3437, 1632, and 1400 cm^−1^ correspond to N−H stretching of amide group, C=O stretching of amide group, and C=O symmetric stretching of COO^−^, respectively [S5, S6].

**
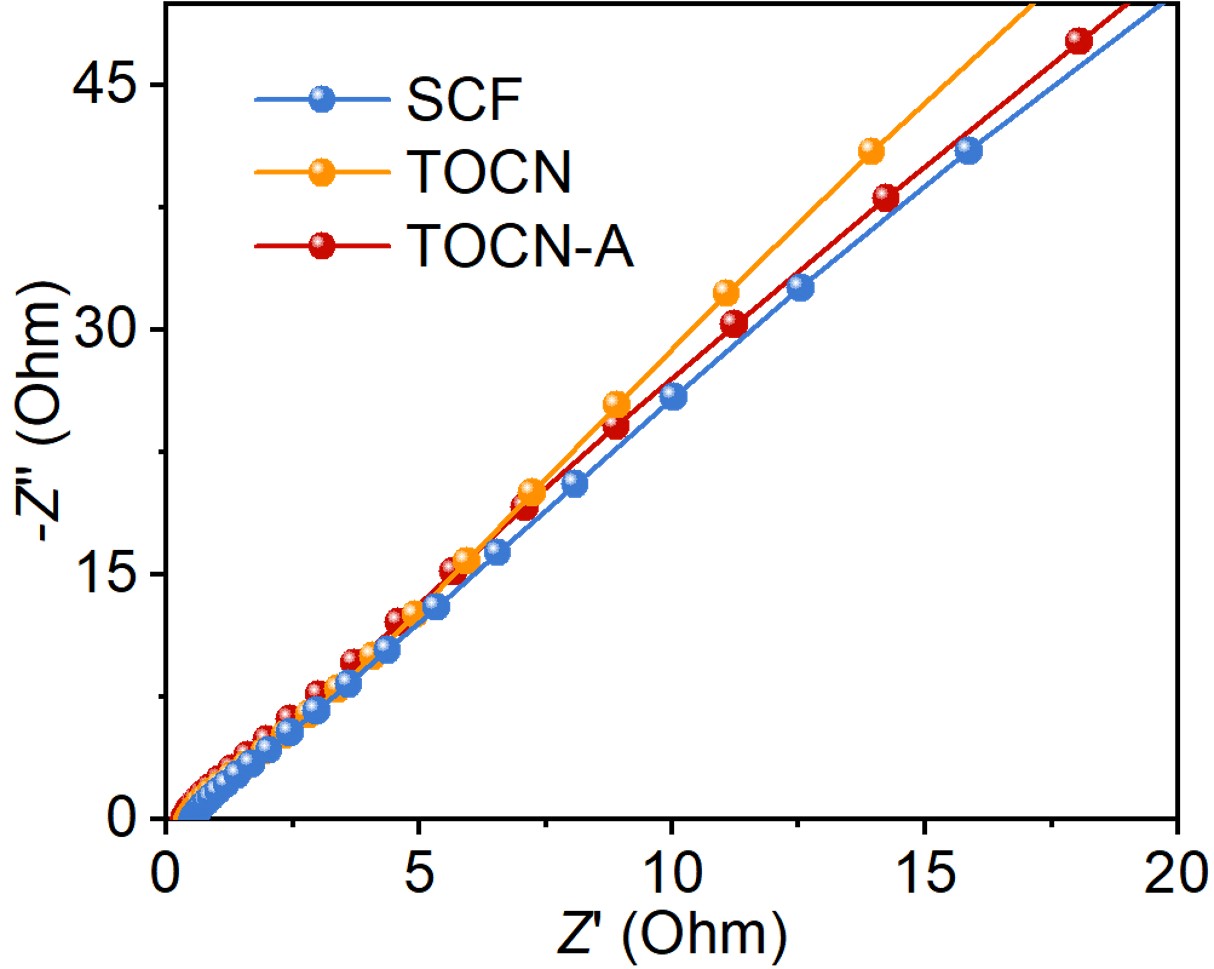
**

**Fig. S8** Nyquist plots of SS//SS cells using SCF, TOCN, and TOCN-A separators


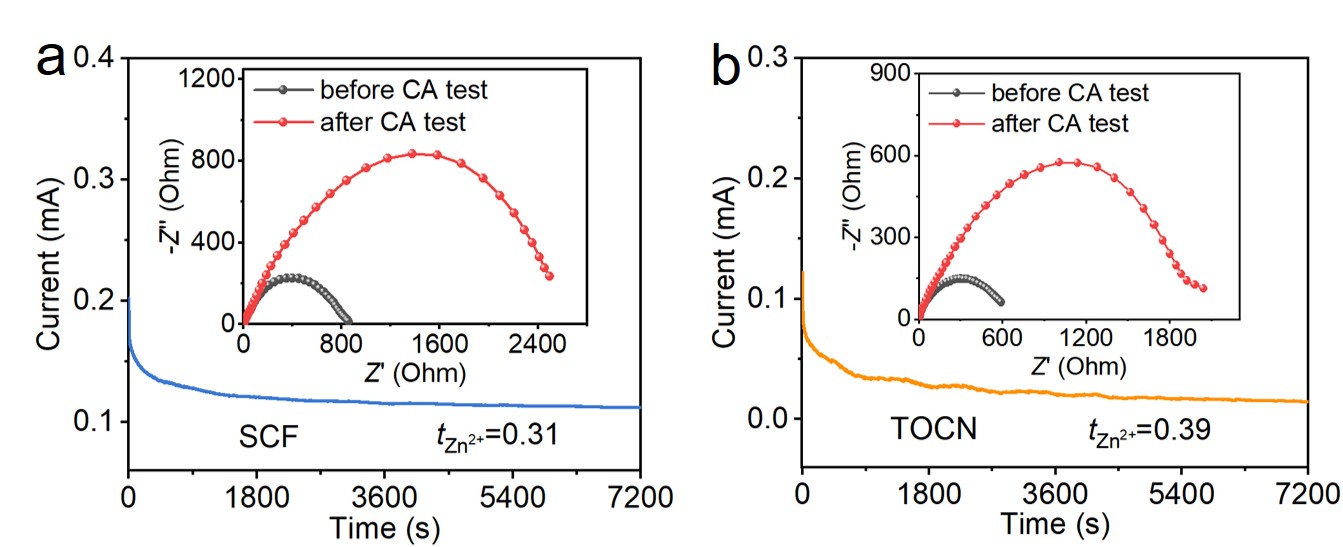


**Fig. S9** CA curves and corresponding Nyquist plots of Zn//Zn cells: **a** using SCF separator and **b** using TOCN separator


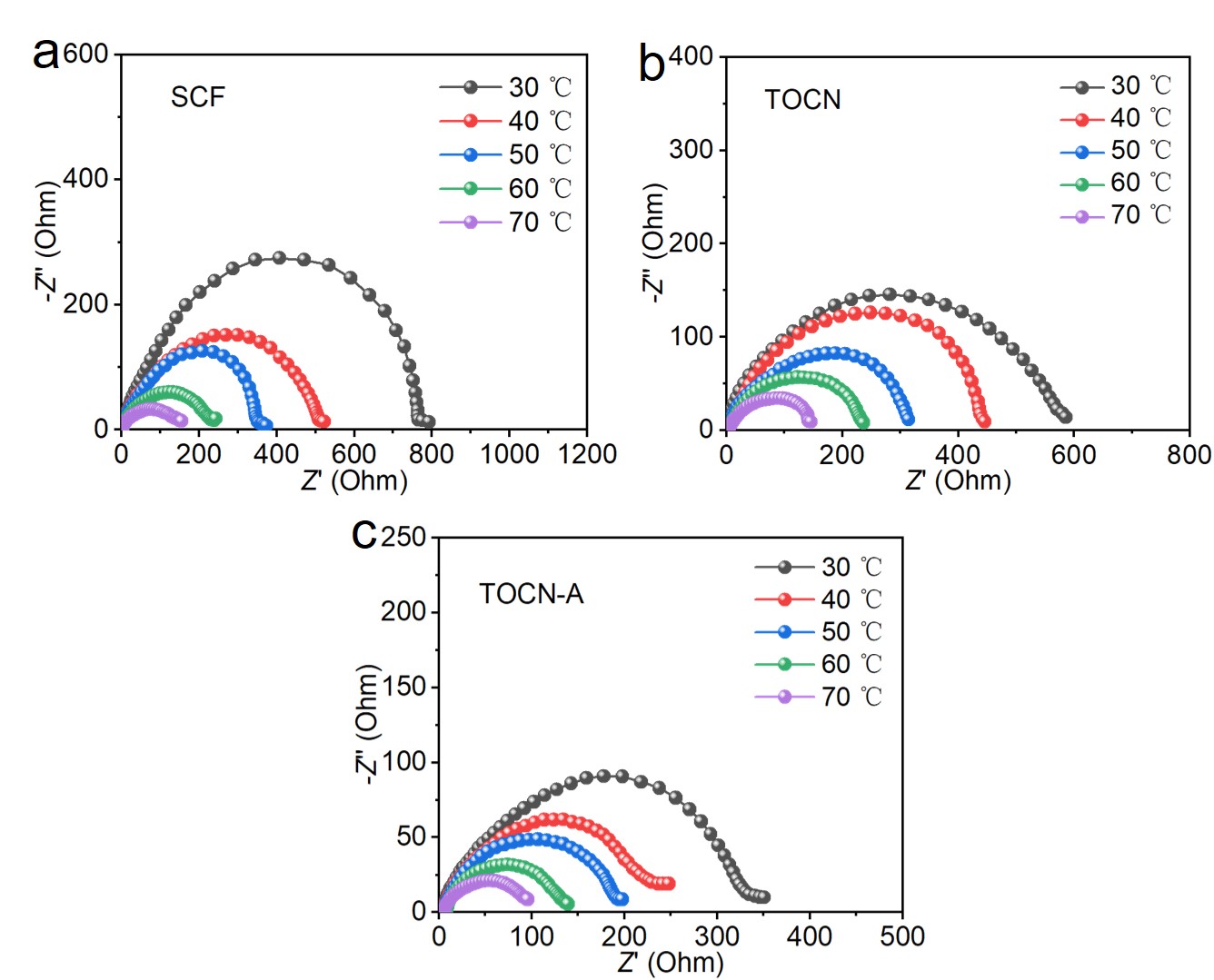


**Fig. S10** Nyquist plots of Zn//Zn cells under different temperatures: **a** using SCF separator, **b** using TOCN separator, and **c** using TOCN-A separator

**
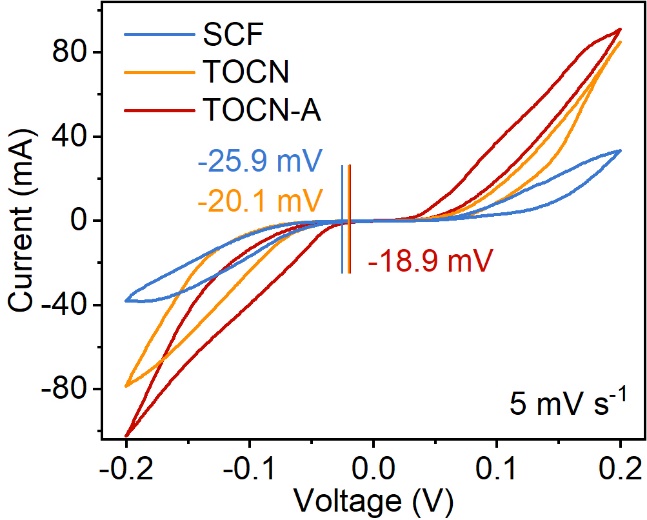
**

**Fig. S11** CV curves of Zn//Zn cells with SCF, TOCN, and TOCN-A separators

**
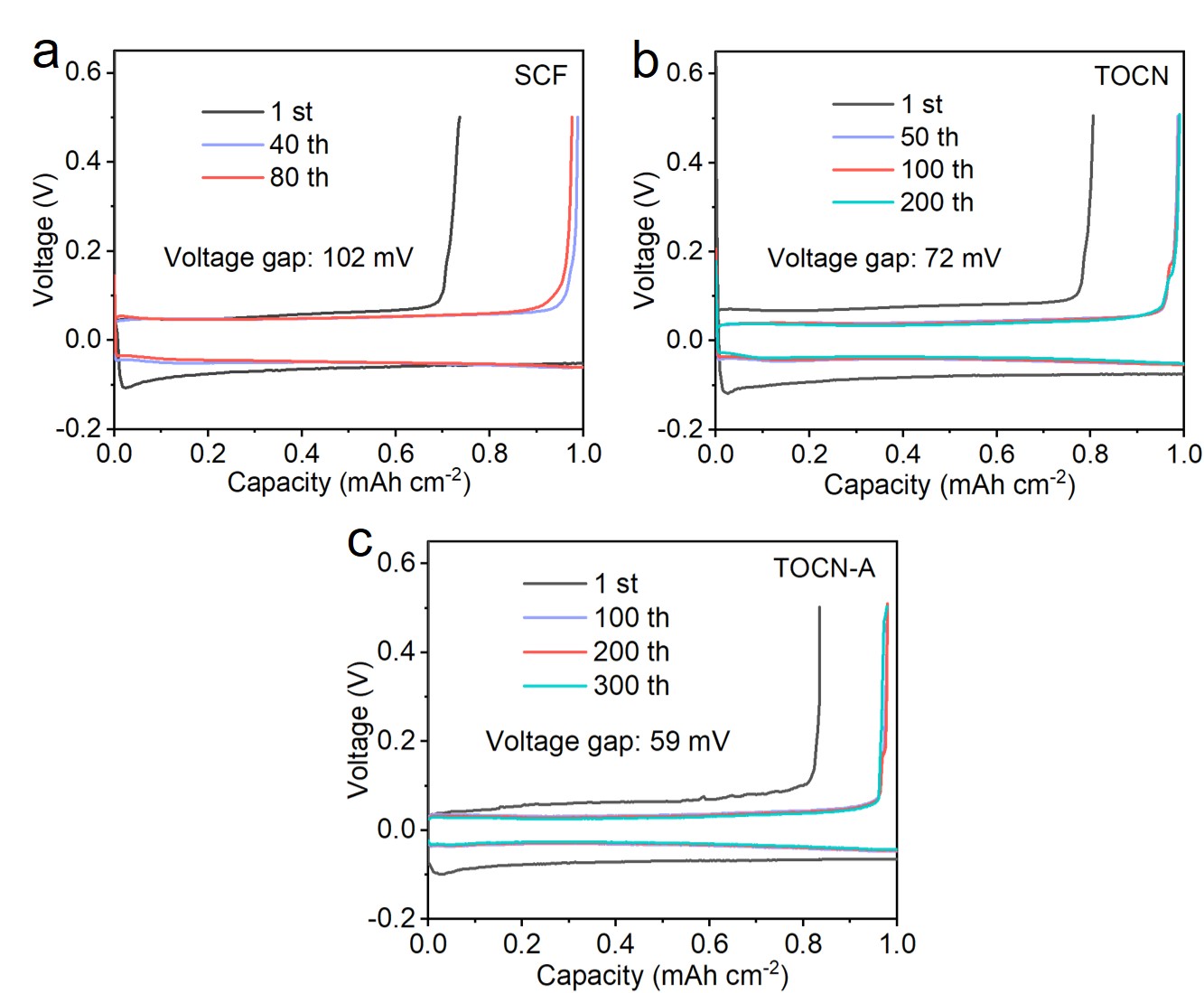
**

**Fig. S12** Voltage-capacity profiles of Zn//Cu cells at different cycles: **a** using SCF separator, **b** using TOCN separator, and **c** using TOCN-A separator

**
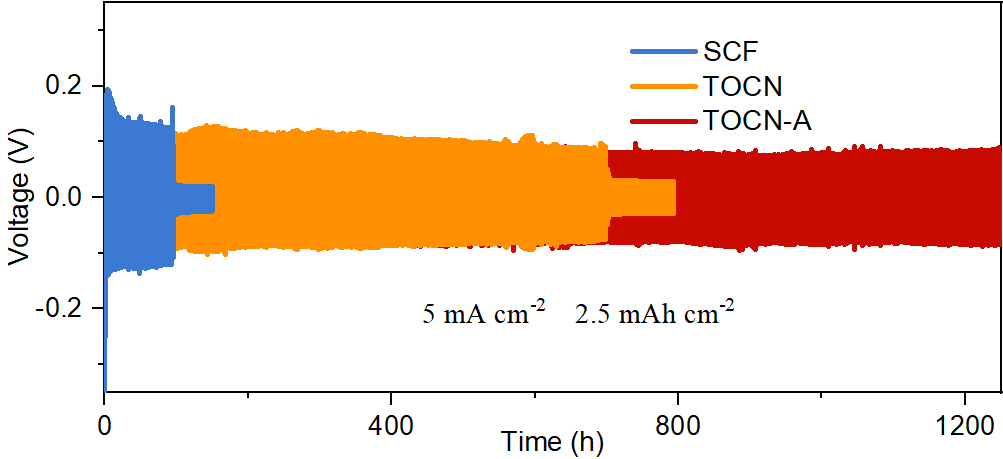
**

**Fig. S13** Cycling performance of Zn//Zn cells with SCF, TOCN, and TOCN-A separators under 5 mA cm^−2^ and 2.5 mAh cm^−2^

**
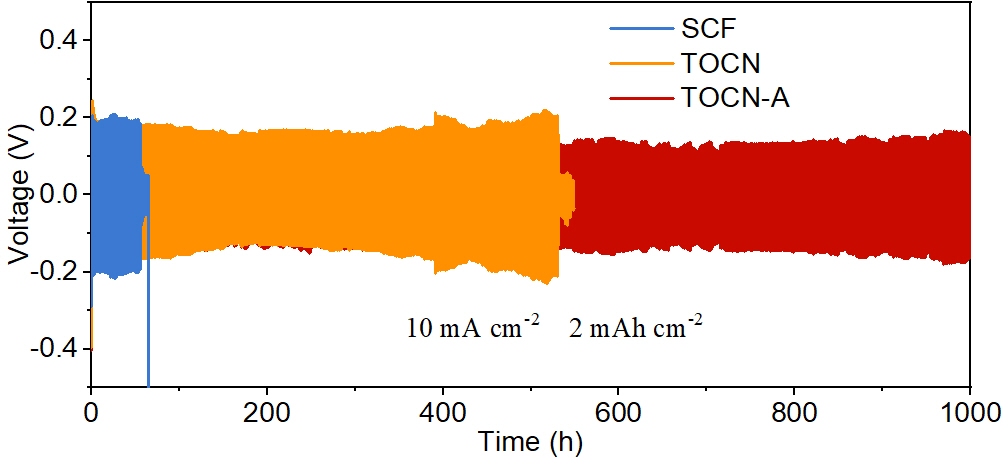
**

**Fig. S14** Cycling performance of Zn//Zn cells with SCF, TOCN, and TOCN-A separators under 10 mA cm^−2^ and 2 mAh cm^−2^


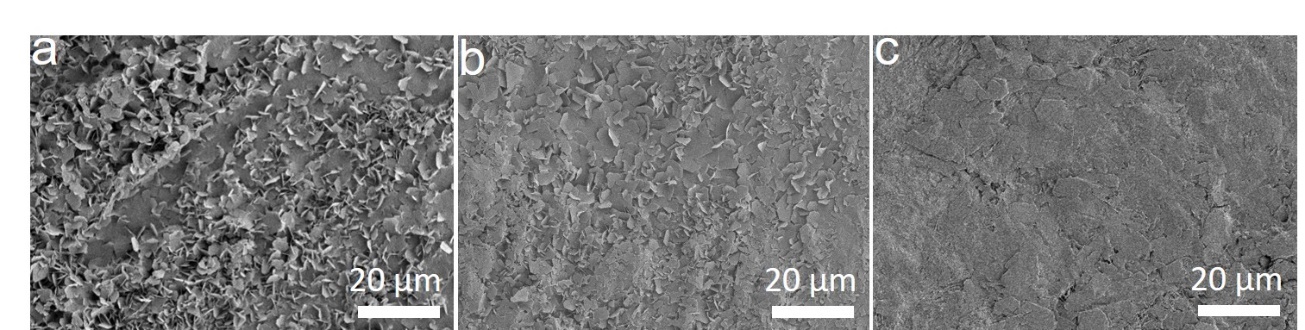


**Fig. S15** Low-magnification SEM images of the Zn electrodes after cycling: **a** using SCF separator, **b** using TOCN separator, and **c** using TOCN-A separator

**
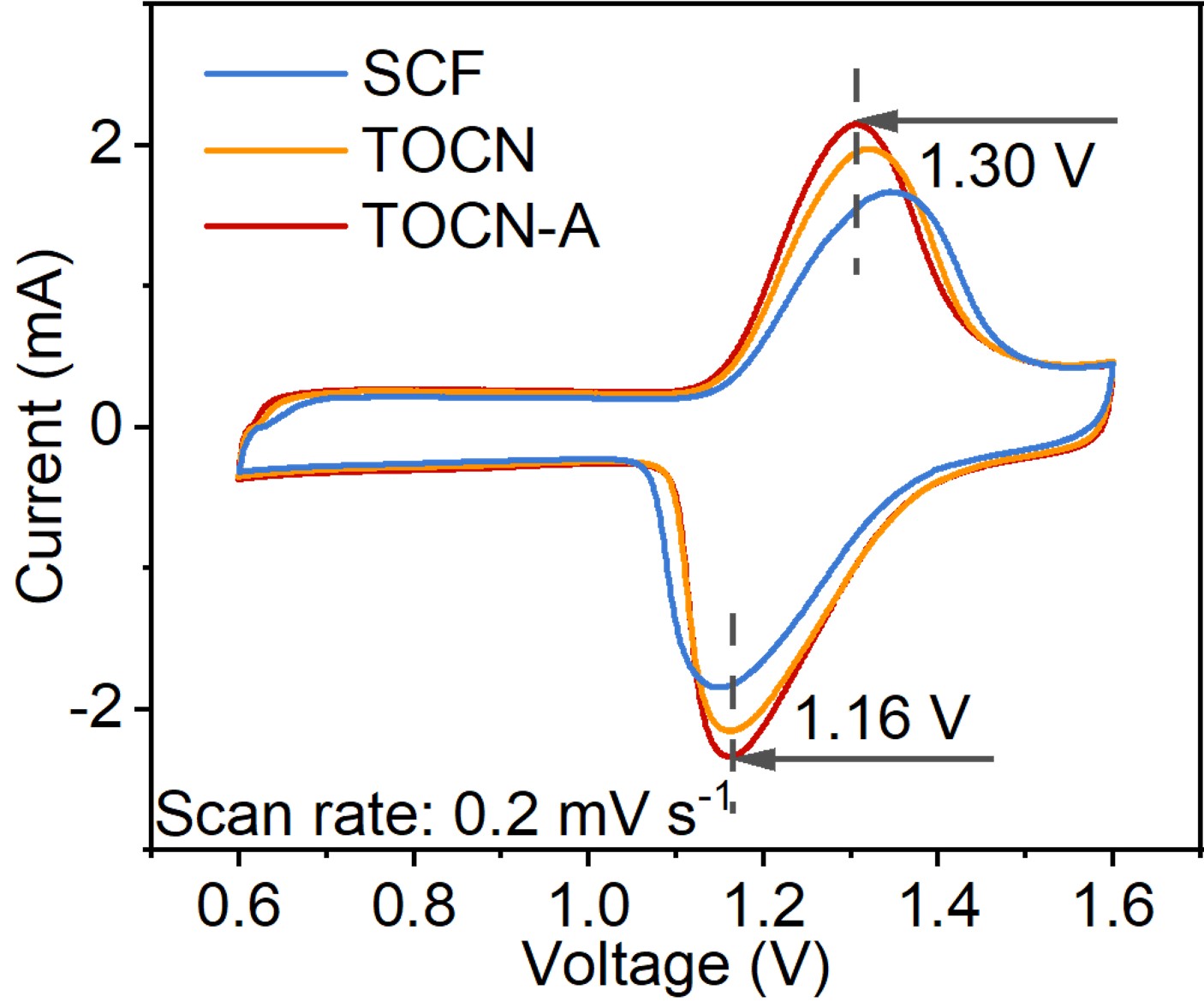
**

**Fig. S16** CV curves of Zn//I_2_ batteries with SCF, TOCN, and TOCN-A separators at a scan rate of 0.2 mV s^−1^


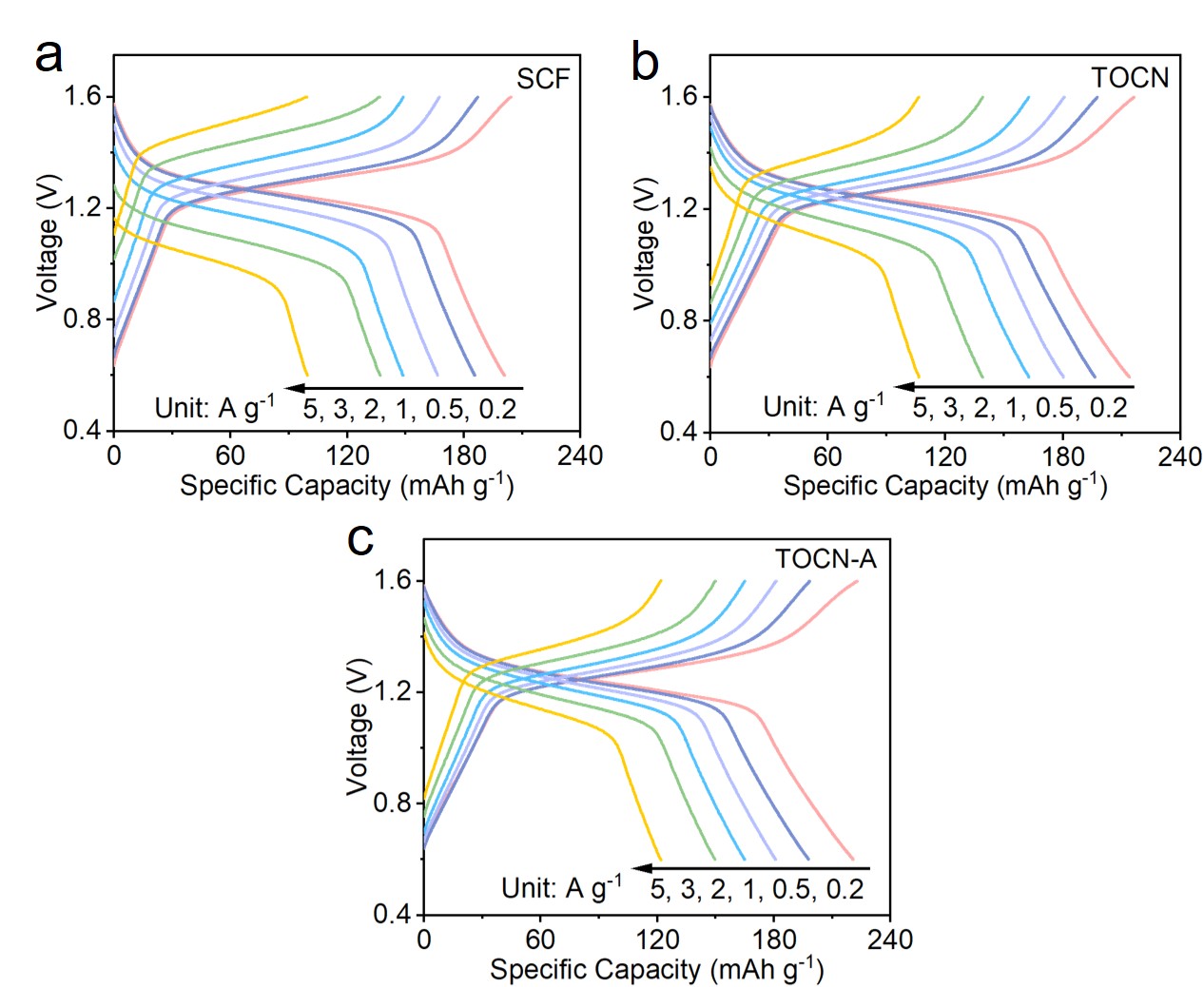


**Fig. S17** GCD profiles of Zn-I_2_ batteries at varying current densities: **a** using SCF separator, **b** using TOCN separator, and **c** using TOCN-A separator

**
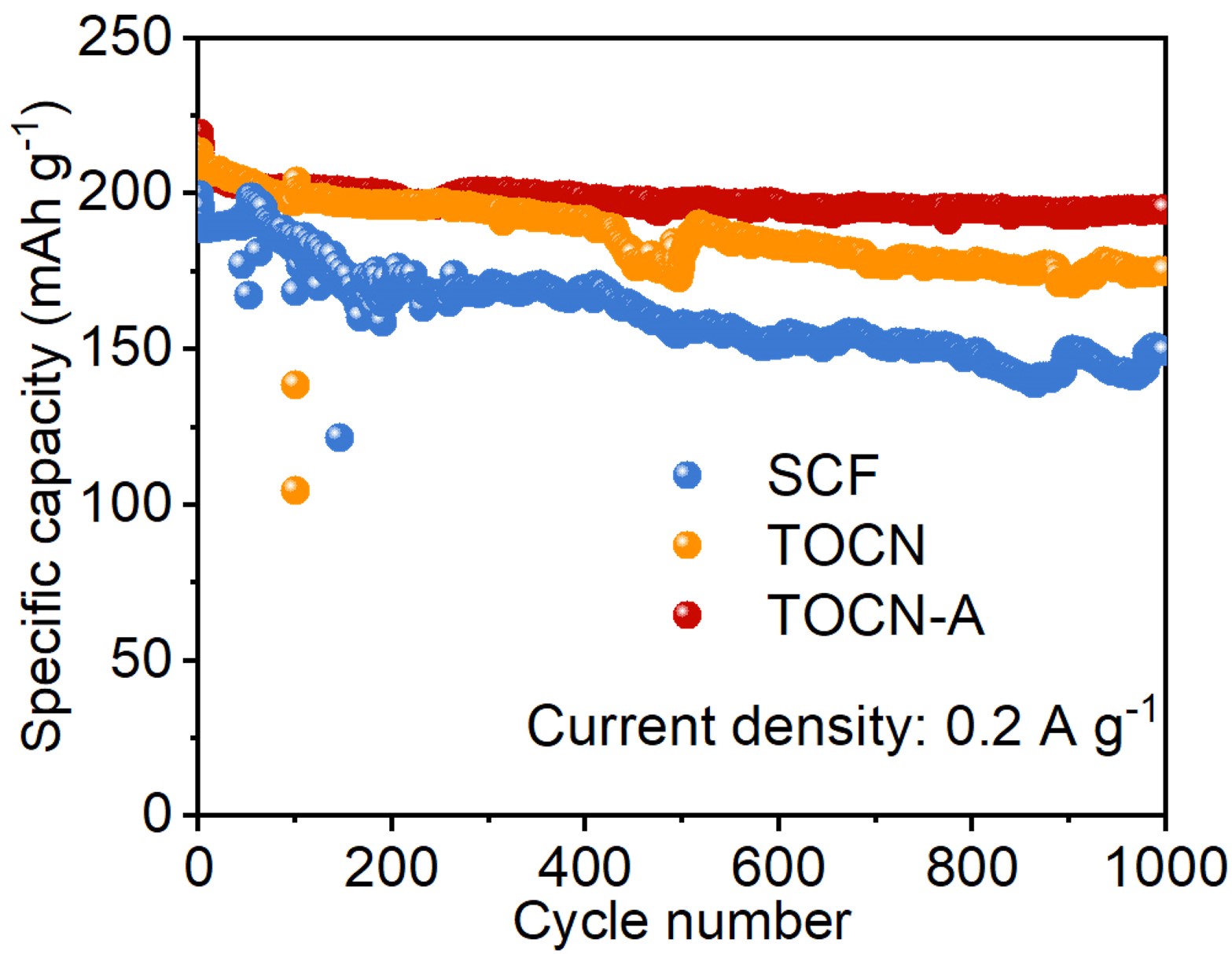
**

**Fig. S18** Cycling performance of Zn//I_2_ batteries with SCF, TOCN, and TOCN-A separators at 0.2 A g^−1^


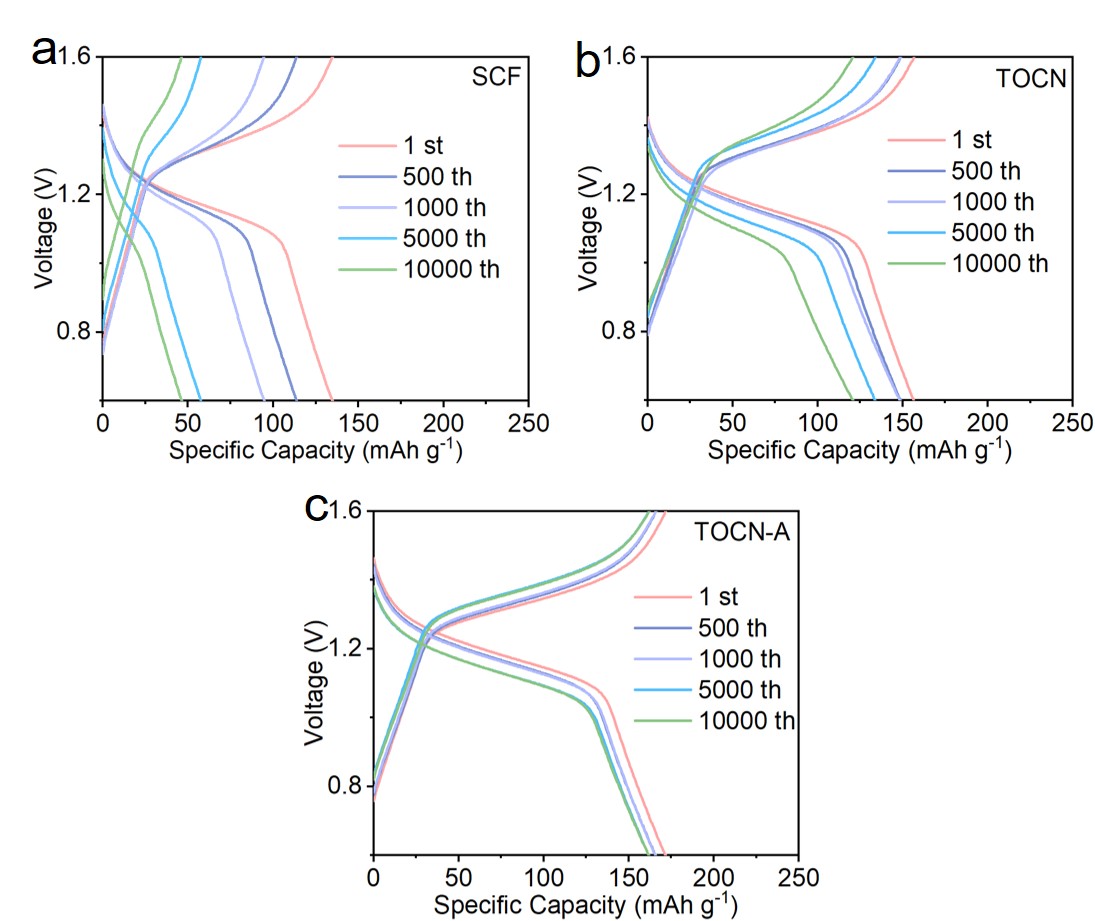


**Fig. S19** GCD profiles of Zn-I_2_ batteries at 2 A g^−1^ and different cycles: **a** using SCF separator, **b** using TOCN separator, and **c** using TOCN-A separator


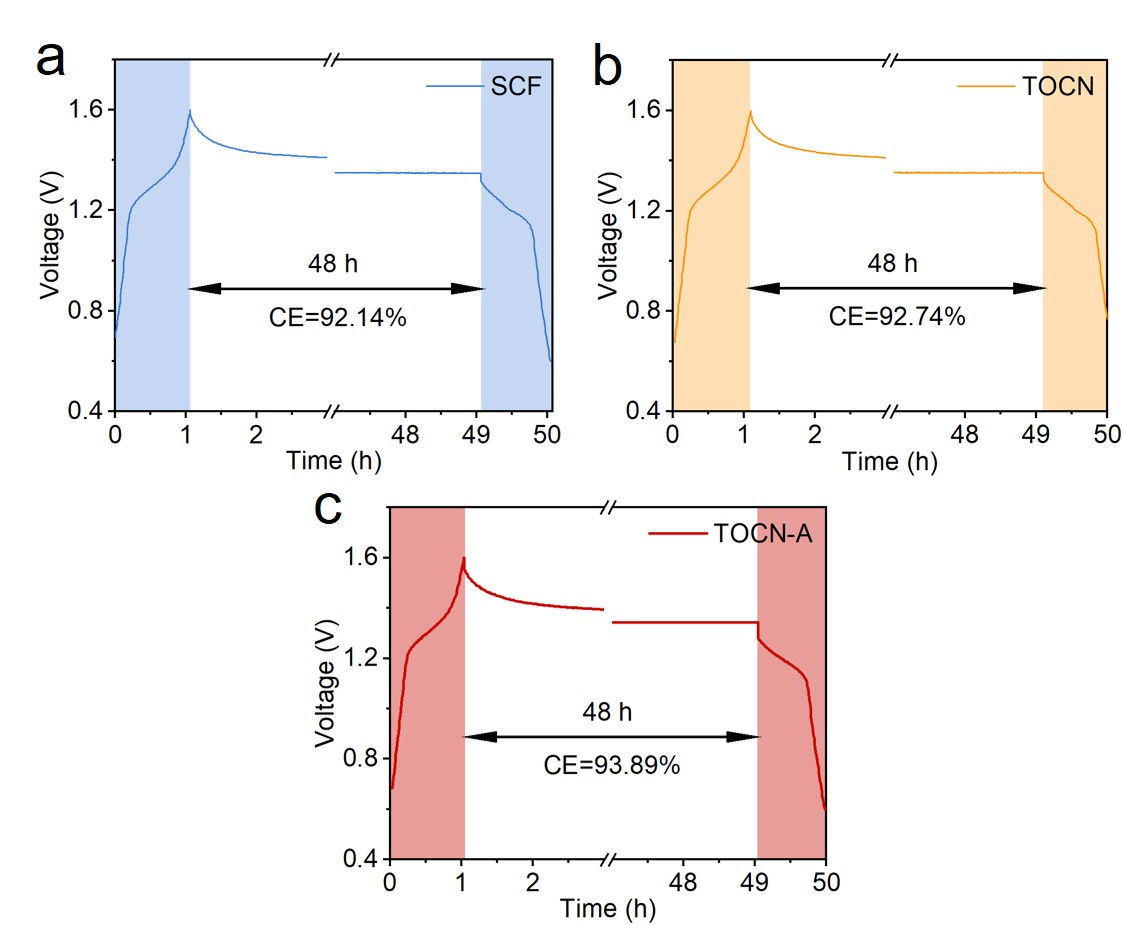


**Fig. S20** Self-discharge behaviors of Zn-I_2_ batteries: **a** using SCF separator, **b** using TOCN separator, and **c** using TOCN-A separator. The batteries were subjected to three GCD cycles at a current density of 0.2 A g^−1^, followed by a 48-h rest period and subsequent discharging at 0.2 A g^−1^

**
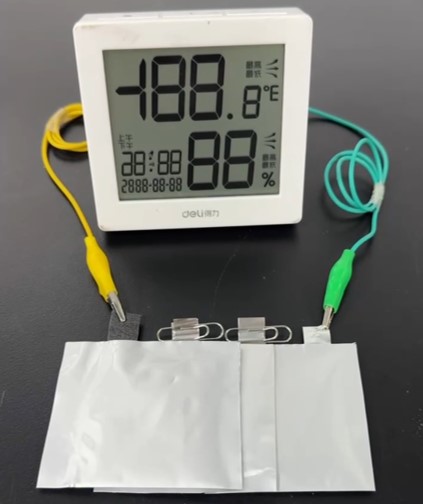
**

**Fig. S21** Photograph of utilizing pouch cells with TOCN-A separator to illuminate an electronic hygrometer

**S4 Supplementary Tables**

**Table S1** Comparison of the thickness and tensile strength of TOCN-A separator and the life span of corresponding Zn//Zn cell in this work with those in previous reports involving various separators for zinc-based batteries. The light blue-shaded zones in the table indicate that nanocellulose-based separators were used in these reports.

| Separator | Thickness | Tensile strength | Current density,  areal capacity | Lifespan | Refs. |
| --- | --- | --- | --- | --- | --- |
| TOCN-A | 20 μm | 147 MPa | 2 mA cm^−2^, 2 mAh cm^−2^  5 mA cm^−2^, 2.5 mAh cm^−2^  10 mA cm^−2^, 2 mAh cm^−2^ | 1800 h  1250 h  1000 h | This work |
| Zr-CNF | 40 μm | − | 10 mA cm^−2^, 2 mAh cm^−2^ | ~710 h | [S7] |
| CCM | 21 μm | 65 MPa | 5 mA cm^−2^, 1 mAh cm^−2^ | 600 h | [S8] |
| HC | 80 μm | − | 4 mA cm^−2^, 2 mAh cm^−2^ | 875 h | [S9] |
| CTNF | 28 μm | 75 MPa | 2 mA cm^−2^, 2 mAh cm^−2^ | 900 h | [S10] |
| M-3-separator | − | − | 1 mA cm^−2^, 1 mAh cm^−2^ | 735 h | [S11] |
| TN-5 | 45 μm | − | 5 mA cm^−2^, 2.5 mAh cm^−2^ | 1000 h | [S12] |
| WCCNF | 70 μm | − | 5 mA cm^−2^, 1.25 mAh cm^−2^ | 1000 h | [S13] |
| FCNF | 23 μm | 121 MPa | 5 mA cm^−2^, 2.5 mAh cm^−2^ | 660 h | [S14] |
| BM | 50 μm | 0.32 MPa | 1 mA cm^−2^, 1 mAh cm^−2^ | 1000 h | [S15] |
| C_3_N_4_@GF | >300 μm | 41.5 MPa | 10 mA cm^−2^, 2 mAh cm^−2^ | 830 h | [S16] |
| PTFE | 50 μm | 34.1 | 10 mA cm^−2^, 2 mAh cm^−2^ | 900 h | [S17] |
| HDP | 250 μm | 6.33 MPa | 1 mA cm^−2^, 1 mAh cm^−2^ | 1500 h | [S18] |
| PAN@SBMA | 72 μm | 13 MPa | 1 mA cm^−2^, 1 mAh cm^−2^ | 1700 h | [S19] |
| SHM | 300 μm | 6.18 MPa | 1 mA cm^−2^, 1 mAh cm^−2^ | 1600 h | [S20] |
| PEM | 160 μm | 7 MPa | 10 mA cm^−2^, 1 mAh cm^−2^ | 380 h | [S21] |

**Supplementary References**

1. G. Kresse, J. Furthmuller, Efficient iterative schemes for ab initio total-energy calculations using a plane-wave basis set. Phys. Rev. B **54**(16), 11169−11186 (1996). <https://doi.org/10.1103/PhysRevB.54.11169>
2. J.P. Perdew, K. Burke, M. Ernzerhof, Generalized gradient approximation made simple. Phys. Rev. Lett. **77**(18), 3865−3868 (1996). <https://doi.org/10.1103/PhysRevLett.77.3865>
3. P.E. Blöchl, Projector augmented-wave method. Phys. Rev. B **50**(24), 17953−17979 (1994). <https://doi.org/10.1103/PhysRevB.50.17953>
4. S. Grimme, Accurate description of van der Waals complexes by density functional theory including empirical corrections. J. Comput. Chem. **25**(12), 1463−1473 (2004). <https://doi.org/10.1002/jcc.20078>
5. Y. Wu, Z. Jiang, Y. Wang, X. Jiang, J. Hou et al., TEMPO oxidized cellulose nanofiber-reinforced sodium alginate encapsulated poly(acrylamide) microcapsules and its releasing behaviours for enhancing oil recovery. Int. J. Biol. Macromol. **281**, 135707 (2024). <https://doi.org/10.1016/j.ijbiomac.2024.135707>
6. T. Kurihara, A. Isogai, Mechanism of TEMPO-oxidized cellulose nanofibril film reinforcement with poly(acrylamide). Cellulose **22**(4), 2607−2617 (2015). <https://doi.org/10.1007/s10570-015-0680-5>
7. S. Yang, Y. Zhang, Y. Zhang, J. Deng, N. Chen et al., Designing anti-swelling nanocellulose separators with stable and fast ion transport channels for efficient aqueous zinc-ion batteries. Adv. Funct. Mater. **33**(42), 2304280 (2023). <https://doi.org/10.1002/adfm.202304280>
8. Y. Zhang, Z. Liu, X. Li, L. Fan, Y. Shuai et al., Loosening zinc ions from separator boosts stable Zn plating/striping behavior for aqueous zinc ion batteries. Adv. Energy Mater. **13**(42), 2302126 (2023). <https://doi.org/10.1002/aenm.202302126>
9. M. Wang, Z. Dai, C. Yang, D. Xu, X. Zhang et al., Boosting de-solvation *via* halloysite nanotubes-cellulose composite separator for dendrite-free zinc anodes. Mater. Today Energy **46**, 101736 (2024). <https://doi.org/10.1016/j.mtener.2024.101736>
10. W. Yang, W. Yang, Y. Huang, Y. Wu, X. Ma et al., Stable Zn anodes enabled by all-cellulose separators with synergistic hydroxyl and carboxyl chemistry. Energy Storage Mater. **80**, 104436 (2025). <https://doi.org/10.1016/j.ensm.2025.104436>
11. T. Zhao, S. Nie, P. Xiao, S. Peng, J. Chen et al., Exploring the influence of MIL-101(Cr) morphologies on the efficacy of cellulose separators for zinc ion battery performance. J. Membrane Sci. **720**, 123788 (2025). <https://doi.org/10.1016/j.memsci.2025.123788>
12. J. Niu, J. Cao, X. Zhang, D. Zhang, C. Yang et al., Titanium nitride-cellulose nanofiber composite separator for Zn anode stability in aqueous batteries. ACS Appl. Energy Mater. **7**(17), 7496−7504 (2024). <https://doi.org/10.1021/acsaem.4c01790>
13. P. Woottapanit, C. Yang, J. Cao, W. Limphirat, S. Saneewons na ayuttaya et al., Inhibition of zinc dendrite growth by WC-cellulose separators for high-performance zinc-ion batteries. ACS Appl. Energy Mater. **6**(20), 10578−10584 (2023). <https://doi.org/10.1021/acsaem.3c01724>
14. Y. Li, X. Peng, X. Li, H. Duan, S. Xie et al., Functional ultrathin separators proactively stabilizing zinc anodes for zinc-based energy storage. Adv. Mater. **35**(18), 2300019 (2023). <https://doi.org/10.1002/adma.202300019>
15. J. Ma, X. Shi, Z. Wang, L. Zhou, X. Liu et al., High-capacity zinc anode enabled by a recyclable biomass bamboo membrane separator. Adv. Mater. **36**(44), 2406429 (2024). <https://doi.org/10.1002/adma.202406429>
16. Z. Luo, L. Ren, Y. Chen, Y. Zhao, Y. Huyan et al., Regulating the interface chemistry of separator to normalize zinc deposition for long lifespan Zn batteries. Chem. Eng. J. **481**, 148448 (2024). <https://doi.org/10.1016/j.cej.2023.148448>
17. G. Wu, R. Zhu, W. Yang, Y. Yang, J. Okagaki, et al., Extension of aqueous zinc battery life using a robust and hydrophilic polymer separator. Adv. Funct. Mater. **34**(33), 2316619 (2024). <https://doi.org/10.1002/adfm.202316619>
18. D. Zhu, Y. Guo, J. Ren, M.A. Abu-Tahon, S.M. El-Bahy et al., A multifunctional and low-cost separator for long-life aqueous Zn metal batteries. Adv. Compos. Hybrid Mater. **7**(6), 205 (2024). <https://doi.org/10.1007/s42114-024-01027-w>
19. L. Cheng, W. Li, M. Li, S. Zhou, J. Yang et al., Zwitterion modified polyacrylonitrile fiber separator for long-life zinc-ion batteries. Adv. Funct. Mater. **34**(48), 2408863 (2024). <https://doi.org/10.1002/adfm.202408863>
20. H. Ao, T. Ma, R. Wang, S. Zhang, Zincophilic and slightly hydrophobic separator with multifunctional groups enables dendrite-free Zn metal anode *via* ion sieving and interfacial confinement effect. Chem. Eng. J. **504**, 159115 (2025). <https://doi.org/10.1016/j.cej.2024.159115>
21. Y. He, R. Zhang, P. Zou, R.W. Chu, R. Lin et al., Polyelectrolyte membrane enables highly reversible zinc battery chemistry *via* immobilizing anion and stabilizing water. J. Am. Chem. Soc. **147**(8), 6427−6438 (2025). <https://doi.org/10.1021/jacs.4c12409>
